# Supplementary material for: Single molecule spectrum dynamics imaging with 3D target-locking tracking
Source: Nat Commun. 2025 Sep 30;16:8686. doi: 10.1038/s41467-025-63787-3 (PMC12485062; doi:10.1038/s41467-025-63787-3)
Supplement: Supplementary file 1 — Supplementary Information File [file 41467_2025_63787_MOESM1_ESM.pdf]

Supplementary Information

Single molecule spectrum dynamics imaging with 3D target-locking tracking

Sha et al.

Section	Page
**Figures**	
Supplementary Figure 1. The impact of defocusing on spectral localization precision.	1
Supplementary Figure 2. Schematic of 3D-SpecDIM setup.	3
Supplementary Figure 3. Schematics of target-locking 3D single-molecule tracking.	4
Supplementary Figure 4. The workflow of the 3D-SpecDIM system.	5
Supplementary Figure 5. Vision Transformer and domain adaptation -based spectral feature recognition	6
Supplementary Figure 6. Comparison of spectral peak emission wavelength recognized methods	7
Supplementary Figure 7. Sequence diagram for camera data acquisition.	8
Supplementary Figure 8. Relationship between exposure time and the number of fluorophores required	9
Supplementary Figure 9. 3D tracking localization precision.	10
Supplementary Figure 10. Single molecule spectral tracking of three distinct molecules.	11
Supplementary Figure 11. Single molecule demonstration with photoblinking.	12

Supplementary Figure 12. Discern two particles switching events during real-time single-particle tracking with 3D-SpecDIM.	13
Supplementary Figure 13. Spectral profile acquisition of 3D-SpecDIM enables high precision ratiometric fluorescence imaging by spectral unmixing	14
Supplementary Figure 14. Mitochondrial size analysis during mitophagy.	16
Supplementary Figure 15. Monitor the mitophagy process with confocal microscopy	17
Supplementary Figure 16. Domain structure of 2×COX8-mGold-HaloTag used in mitophagy experiments	18
Supplementary Figure 17 pH titration of mGold and JF549 dye	19
Supplementary Figure 18. Comparison of the 3D-SpecDIM-enabled spectral unmixing ratiometric fluorescence detection and the conventional dual-channel ratiometric detection	20
Supplementary Figure 19. Spectrum dynamics tracking of pH-sensitive probe labeled mitochondrion in cells without mitophagy	21
Supplementary Figure 20. The histogram distribution of laser irradiation time for blebbing and no blebbing events	22
Supplementary Figure 21. Characterize the polarity of lipids membrane with 3D-SpecDIM	23

Supplementary Figure 22. The relationship between spectral peak emission wavelength of Nile Red and ET(30)	24
Supplementary Figure 23. Signal to background ratio characterization of AgNPs scattering imaging in cell and the bright field images of cellular blebbing process	25
Supplementary Figure 24. 3D trajectories of AgNPs during celluar blebbing	26
Supplementary Figure 25. Spectral registration	27
Supplementary Figure 26. Comparison of green fluorescent dead spectra acquired using 3D-SpecDIM (circle) and a commercial spectrometer	29
Supplementary Figure 27. Confocal images of mitochondria and lysosome during mitophagy	30
Supplementary Figure 28. Comparison between 2×cox8-mGold-HaloTag and mitochondrial dyes	31
Supplementary Figure 29. Align the tracking trajectory position with EMCCD image	32
Supplementary Figure 30. The APD signal as a function of time for the relevant data in the main text	33
Supplementary Figure 31. Kymograms for the relevant trajectories in main text.	34
Supplementary Figure 32. Simulated datasets for ViT model training	35

Supplementary Figure 33. Evaluation of QE correction in spectral peak emission wavelength estimation.	36
**Tables**	
Supplementary Table 1. Temporal resolution analysis of 3D-SpecDIM system.	38
Supplementary Table 2. Cell blebbing occurrence analysis under different conditions.	39
Supplementray Tables 3. Statistics data of fluorescence spectral peak emission wavelength changes during cell blebbing.	40
Supplementary Table 4. Optical path configurations and experimental parameters	41
Supplementary Table 5. Preparation of Silicon spheres coated with SLB.	43
Supplementary Table 6. Spectral detection performance comparison between 3D-SpecDIM and Zeiss LSM 980 spectral scanning confocal microscope.	44
Supplementary Table 7. Spectral detection performance comparison between 3D-SpecDIM and Zeiss LSM 980 spectral scanning confocal microscope.	45
**Notes**	
Supplementary Note 1. Silicon spheres coated with supported lipid bilayers.	46

Supplementary Note 2. Vision Transformer and domain adaption-based spectral feature recognition for improving spectral imaging precision.	48
Supplementary Notes 3. Workflow and details of 3D-SpecDIM tracking and spectral data acquisition	53
Supplementary Notes 4. Multi-resolution imaging with 3D-SpecDIM.	57
Supplementary Notes 5. Calculation of diffusion coefficient.	58

Supplementary Figures

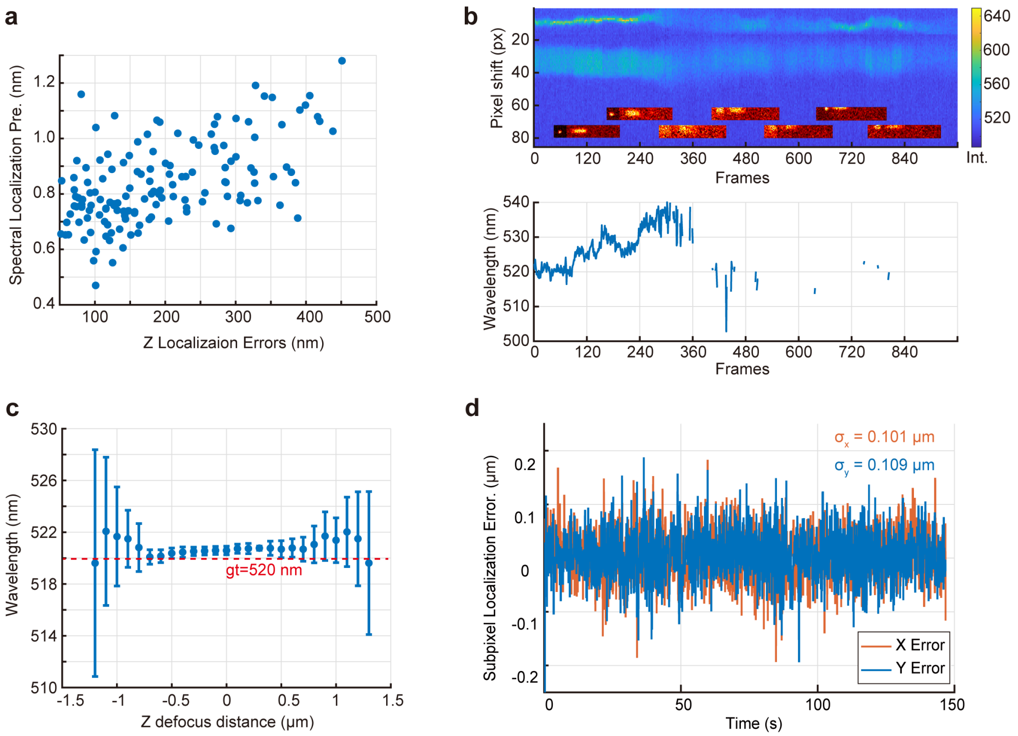

Supplementary Figure 1. **The impact of defocusing on spectral localization precision. (a)** Z-axis localization precision as a function of spectral localization precision. Fixed fluorescent beads were tracked while systematically adjusting the feedback control parameters of the axial piezo stage to modify localization precision. Localization precisions were calculated with 1 second window. **(b)** The spectral images of freely diffusing fluorescent bead in aqueous solution were recorded by 3D-SpecDIM without target-locking tracking. Upper panel: kymograms of spectral images. Lower panel: the spectral peak emission wavelength as a function of frame number, with only successfully fitted data points shown. At certain time points, the signal-to-noise ratio was too low to obtain a valid spectral peak emission wavelength estimation. **(c)** Defocusing distance as a function of spectral peak emission wavelength. A fixed fluorescent particle was imaged at various axial positions. As the Z defocusing distance increased from 0 to ±1.2 μm, the spectral localization precision decreased from

0.6 nm to approximately 2.6 nm at a Z defocusing distance of -1.2 μm and 2.1 nm at 1.2 μm. **(d)** Impact of motion blur on subpixel positional precision of EMCCD image with target-locking tracking. Data represents the trajectories shown in Fig. 2a-c. Errors were calculated by subtracting the average x- and y-pixel localization values across all frames from the x- and y-localization values in each individual EMCCD frame.

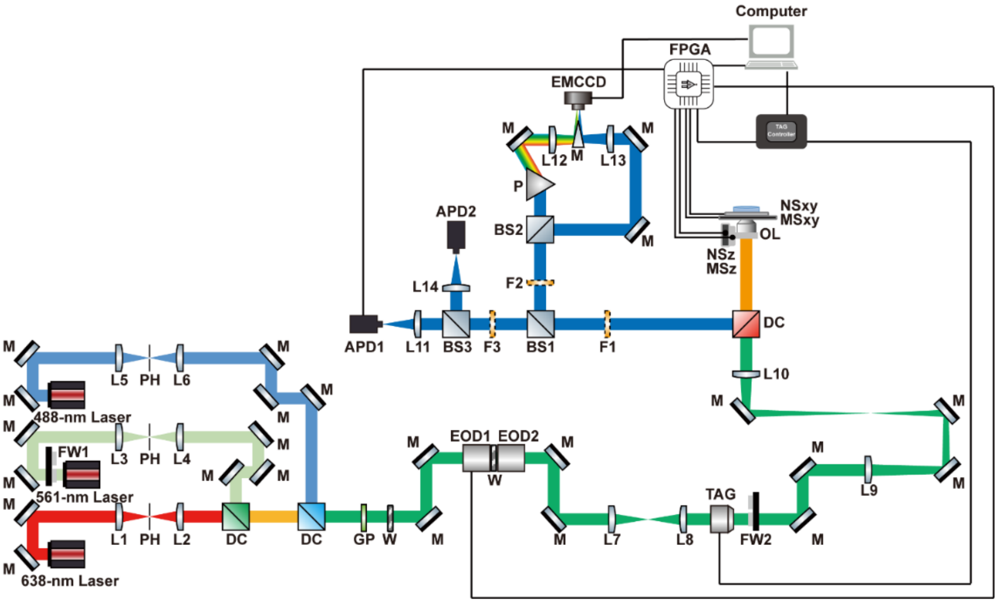

Supplementary Figure 2. Schematic of 3D-SpecDIM setup. M: mirror; L: lens; PH: pinhole; FW: filter-wheel; PH: pinhole; DC: dichroic mirror; GP: Glan-Thompson polarizer; W: half waveplate; EOD: electro-optic deflector; TAG: TAG lens, OL: objective lens; MSxy: xy microstage; MSz: z microstage; NSxy: xy nanopositioner stage; NSz: z nanopositioner stage; FPGA: field programmable gate arrays; F: Fluorescence filter; BS: beam splitter; APD: avalanche diode detector; P: prism.

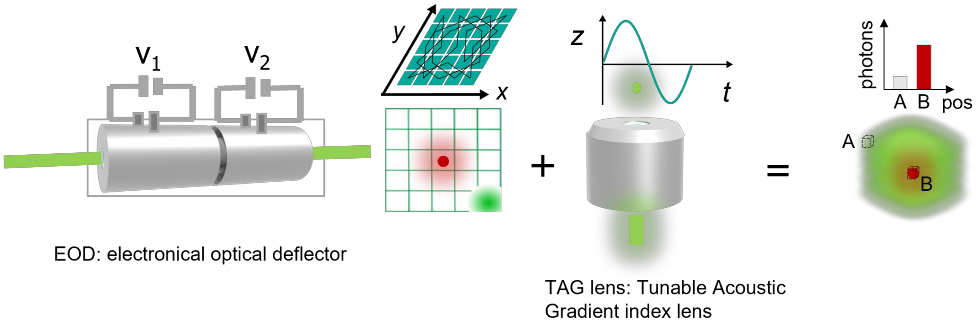

Supplementary Figure 3. Schematics of target-locking 3D single-molecule tracking. The laser focus is deflected in three dimensions by the EOD and TAG lens. The arrival times of fluorescence photons detected by the APD are utilized for real-time position estimation using an FPGA. Subsequently, an active feedback control signal is applied to the piezo stage to maintain the target fluorophore within the excitation volume.

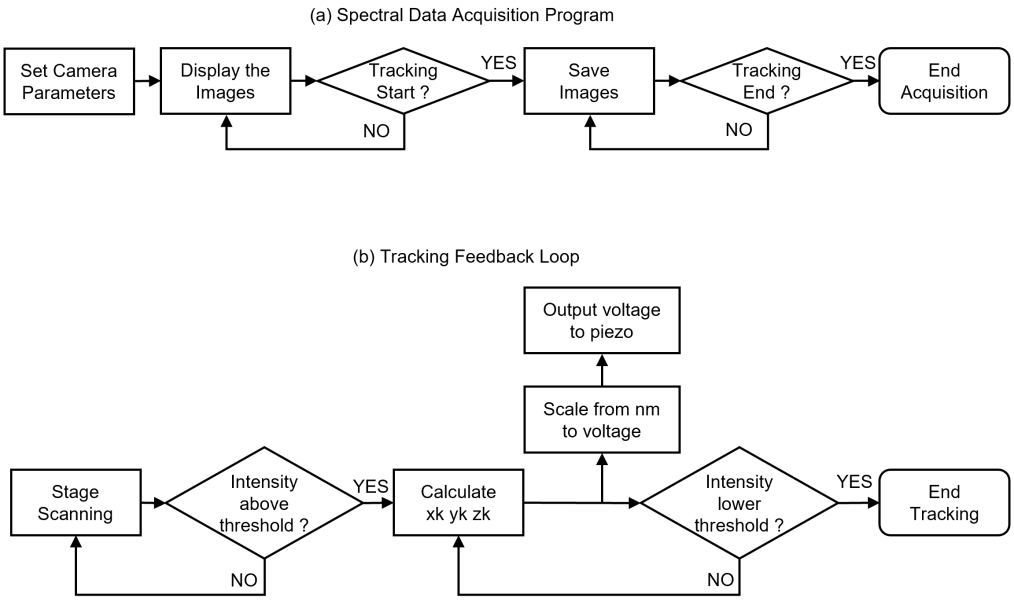

Supplementary Figure 4. The workflow of the 3D-SpecDIM system. (a) Spectral data acquisition program. Real-time EMCCD images are displayed via a custom LabVIEW program, which automatically saves spectral images throughout the tracking session. **(b)** Spectral tracking feedback loop. The system tracks particles by updating positional estimates, converting them to voltage signals to control the piezo stage, and recording the piezo stage positions and spectral images.

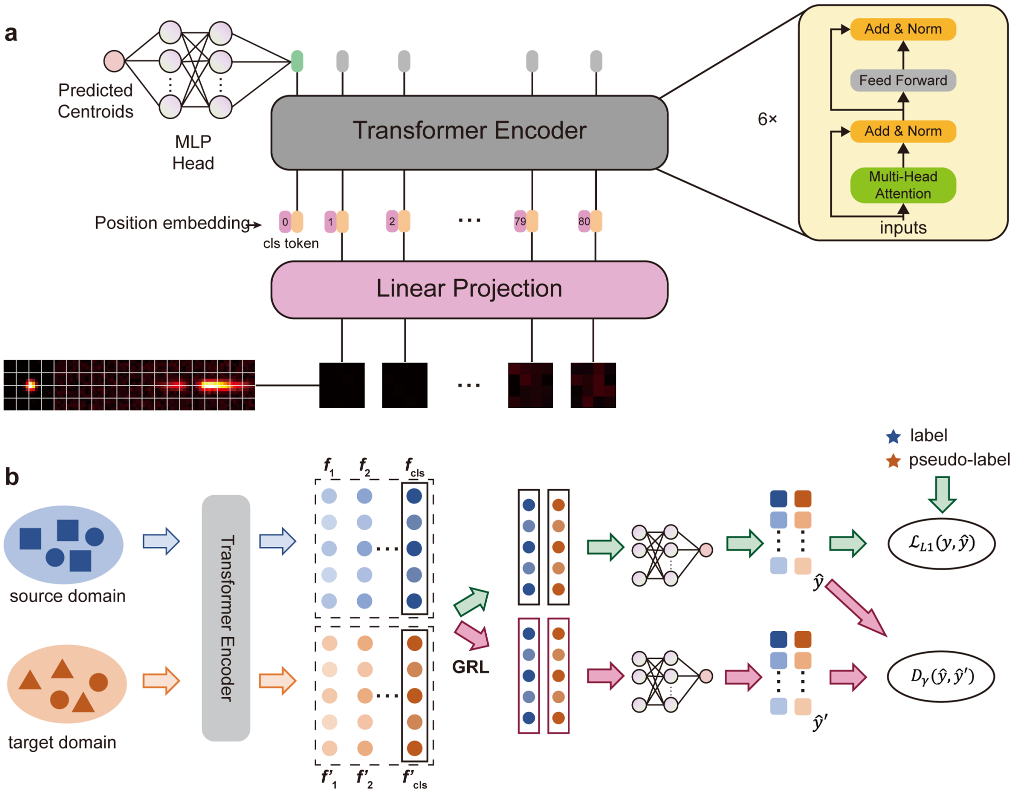

Supplementary Figure 5. Vision Transformer and domain adaptation-based spectral feature recognition. (a) Schematic of training process. Images with size of 16×80 are segmented into 4×20 patches using a 4×4 patch size, followed by the addition of learnable positional encodings after a linear projection layer. Several MLA (Multilayer Attention) layer iterations yield an output head, then spectral peak emission wavelength features derived from a single-layer MLP (Multilayer Perceptron). **(b)** Inference process based on domain adaptive algorithm. For data that is unlabeled and falls outside the training data distribution, the system employs a gradient reversal layer (GRL) along with Margin Disparity Discrepancy ($D_\gamma(\hat{y}, \hat{y}')$) loss to finely tune the parameters of the Encoder. See **Supplementary Note 2** for detailed information.

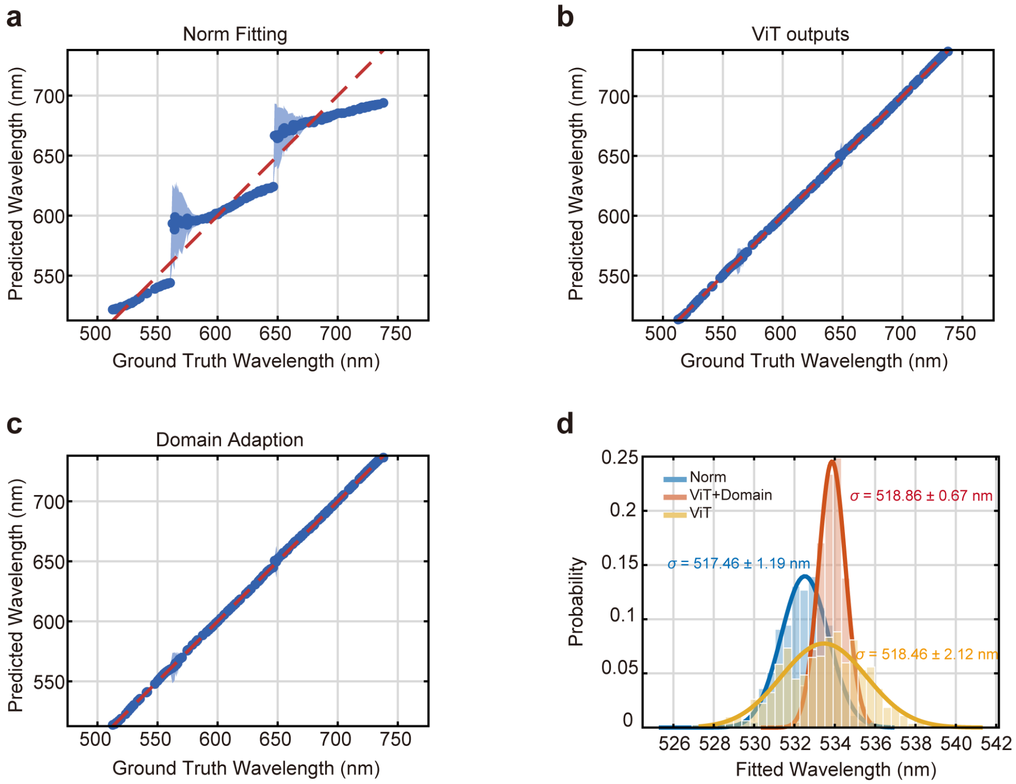

Supplementary Figure 6. Comparison of peak emission wavelength recognized methods. (**a-c**) Comparison the spectral localization precisions of **(a)** traditional normal fitting method, **(b)** Vision Transformer without domain adaption strategy, and **(c)** Vision Transformer with domain adaption strategy on simulation data sets. **(d)** Comparison of spectral localization precision across different methods using fluorescent bead spectra data. The Vision Transformer with domain adaption strategy shows improved spectral precisions.

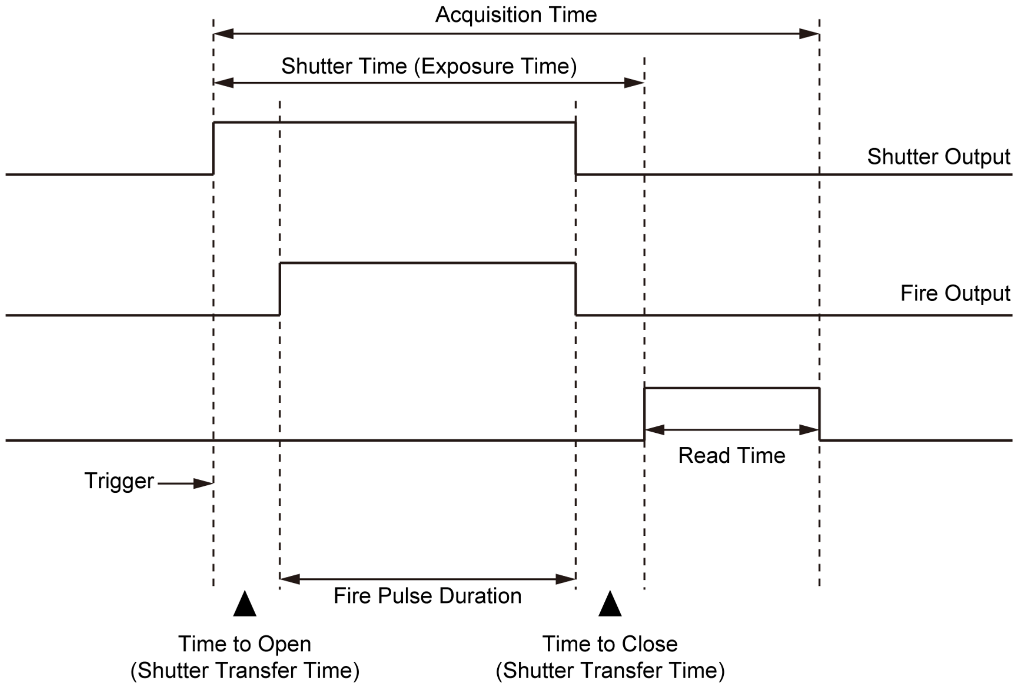

Supplementary Figure 7. Sequence diagram for camera data acquisition. The achievable detection time resolution of the system is primarily determined by the system's hardware capabilities (such as the shutter transfer time and readout time) without considering the photon budget from the sample.

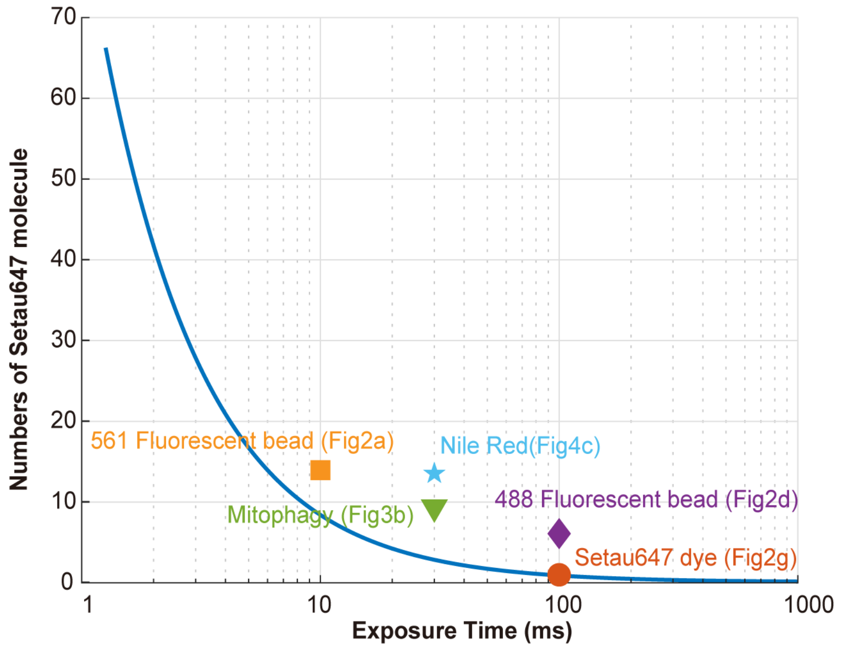

Supplementary Figure 8. The relationship between exposure time and the number of SeTau 647 fluorophores required to achieve a minimum spectral localization error of 5 nm. The blue solid line represents a theoretical benchmark, obtained by calculating the photon counts required to reach 5 nm spectral precision at selected exposure times (50 ms, 100 ms, 200 ms, and 500 ms), and fitting these points with an inverse function. Data points represent experimental configurations used in this paper, including different dyes and exposure times. All fluorescence intensities were normalized and converted to the equivalent number of SeTau 647 molecules.

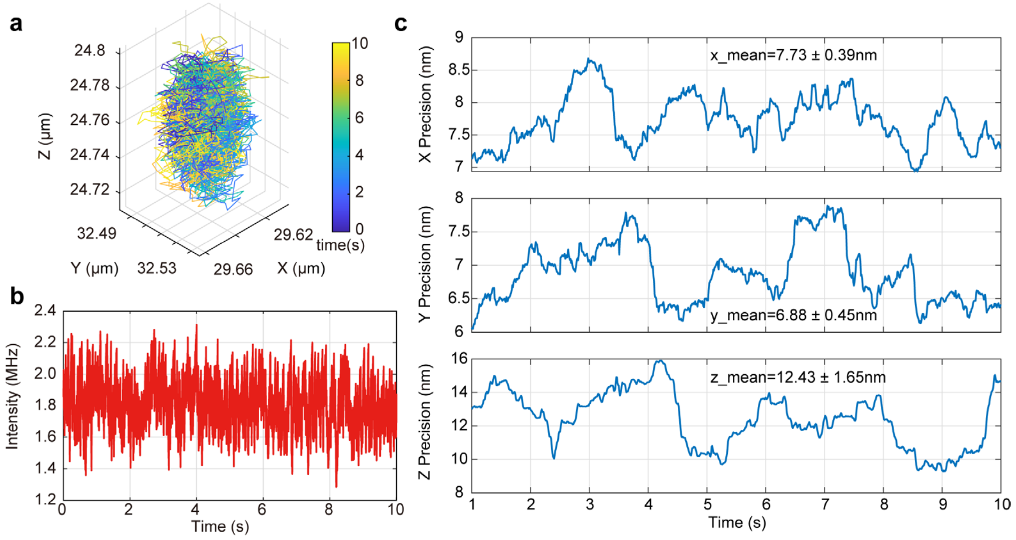

Supplementary Figure 9. 3D tracking localization precision. (**a**) 3D trajectory of a fixed 200 nm fluorescence bead. (**b**) Fluorescence intensity as a function of time. (**c**) Tracking precision as a function of time for X, Y, and Z, respectively. The precision is measured by the standard deviation of position of 1 s data with 10 ms sliding window.

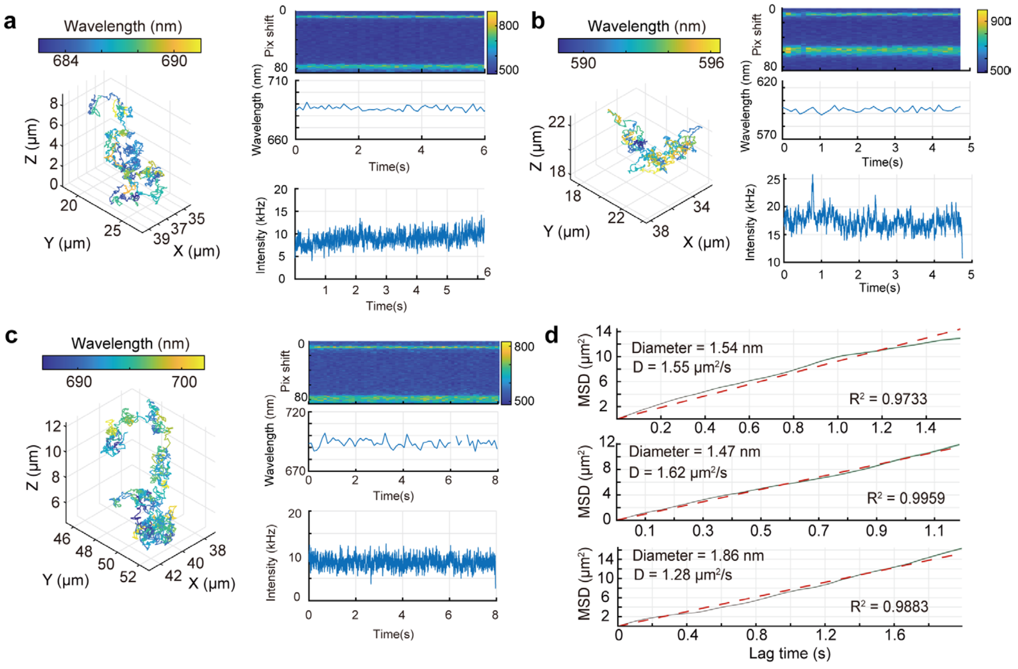

Supplementary Figure 10. Single molecule spectral tracking of three distinct molecules. (**a**) Left: 3D moving trajectory of a single Atto 665 fluorescent molecule diffusing in 90 wt% glycerol. Right: spectral peak emission wavelength (up panel) and intensity (bottom panel) as a function of time. (**b**) Left: 3D moving trajectory of a single Atto 565 fluorescent molecule diffusing in 90 wt% glycerol. Right: spectral peak emission wavelength (up panel) and intensity (bottom panel) as a function of time. (**c**) Left: 3D moving trajectory of a single SeTau 647 fluorescent molecule diffusing in 90 wt% glycerol. Right: spectral peak emission wavelength (up panel) and intensity (bottom panel) as a function of time. (**d**) Mean square displacement (MSD) of Atto 665 (top), Atto 565 (middle), and Setau 647 (bottom) as a function of lag time for trajectory (**a-c**).

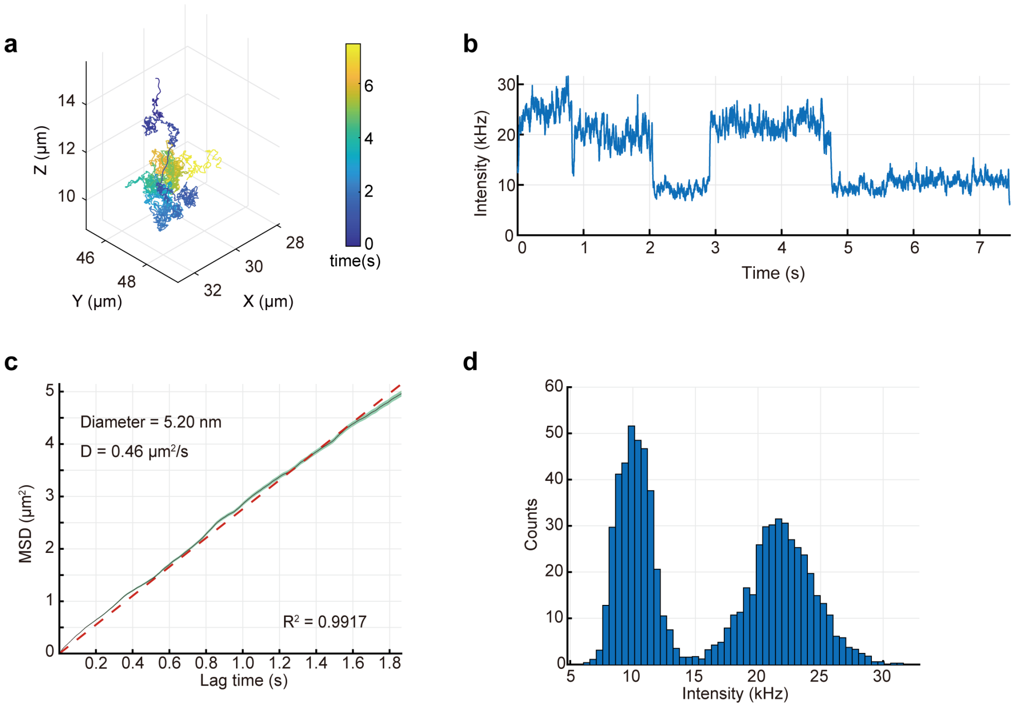

Supplementary Figure 11. Single molecule demonstration with photoblinking. (a) 3D trajectory of a two-Atto 565-labeled BSA protein diffusing in a 90 wt.% glycerol solution, with the trajectory color-coded to represent time. (**b**) Fluorescence intensity as a function of time for the trajectory shown in (**a**), demonstrating alternation between single-dye and two-dye intensity levels. A lower laser power was used in this experiment to reduce photobleaching (1.56 μW after objective), resulting in decreased fluorescence intensity compared to Fig. 2m. (**c**) Mean square displacement as a function of lag time for the trajectory in (**a**). (**d**) Intensity distribution histogram for the trajectory shown in (**a**). The histogram displays two distinct peaks: the higher peak corresponds to both dyes being in the emissive state, while the lower peak reflects one dye transitioning to a dark state.

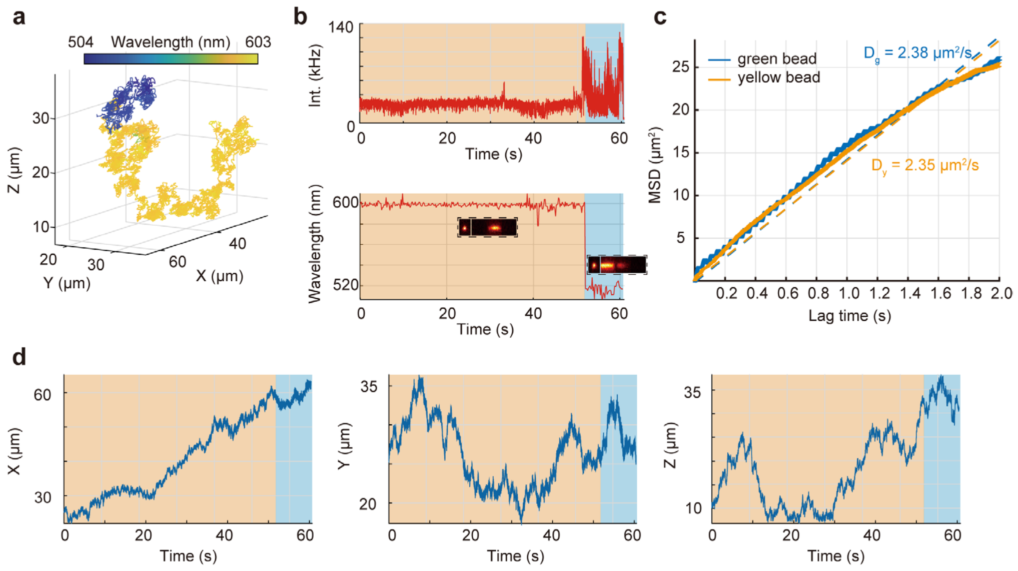

Supplementary Figure 12. Discern two particles switching events during real-time single-particle tracking with 3D-SpecDIM. Green and yellow fluorescent beads were mixed in solution and tracked with 3D-SpecDIM. (**a**) 3D trajectory of a particle switching event. The trajectory of yellow fluorescent particle and green fluorescent particle are encoded with yellow color and blue color, respectively. (**b**) The fluorescence intensity (upper panel) and the fluorescence spectral peak emission wavelength (lower panel) as a function of time in trajectory (**a**). Inserts show the spectrum image in EMCCD. (**c**) The mean square displacements as a function of time for yellow particle and green particle. The diffusion coefficients of them are 2.35 $\mu m^2/s$ and 2.38 $\mu m^2/$, respectively. (**d**) The x, y, and z position as a function of time in trajectory (a).

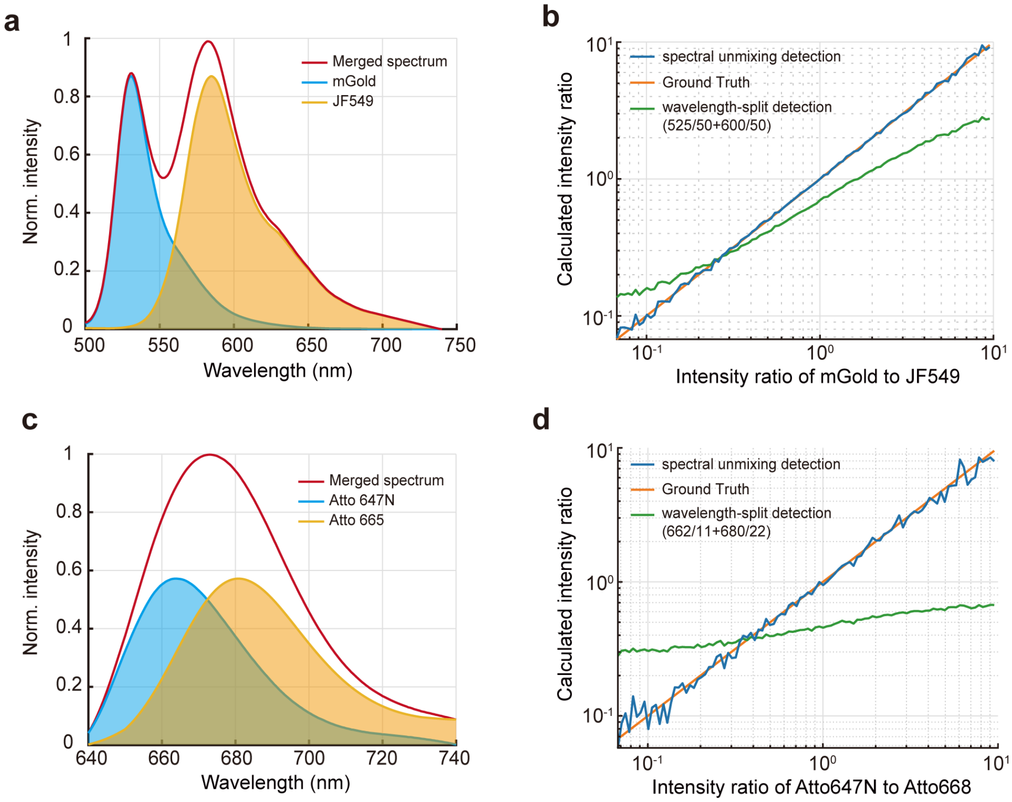

Supplementary Figure 13. Spectral profile acquisition of 3D-SpecDIM enables high precision ratiometric fluorescence imaging by spectral unmixing. (a) The fluorescence emission spectrum of mGold and JF549 dye. The red line is the summed spectral intensity profile with both dyes have the peak intensity of 1. **(b)** Simulated comparison of wavelength-split detection method and 3D-SpecDIM enabled spectral unmixing in ratiometric fluorescence imaging. In wavelength-split detection method, two band pass filters (525/50 and 600/50) were adopted to collect the fluorescence of mGold and JF549 dye. The intensity ratio is calculated with the sum of fluorescence signal within the wavelength range defined by the band pass filters. In spectral unmixing method, fluorescence spectral profiles of mGold and JF549 dye were recovered from the summed spectral profile. The intensity ratio is calculated as the ratio of their spectral profile peak values. We set a various of intensity ratio of mGold to JF549 and compared the wavelength-split detection method (green

line) and spectral unmixing method (blue line). The result shows that the 3D-SpecDIM enabled spectral unmixing method demonstrated pronounced precision enhancement. (**c**, **d**) Similar analysis with (**a**, **b**) but using a larger spectrum-overlapping fluorescent dye pair, Atto 647N and Atto 665.

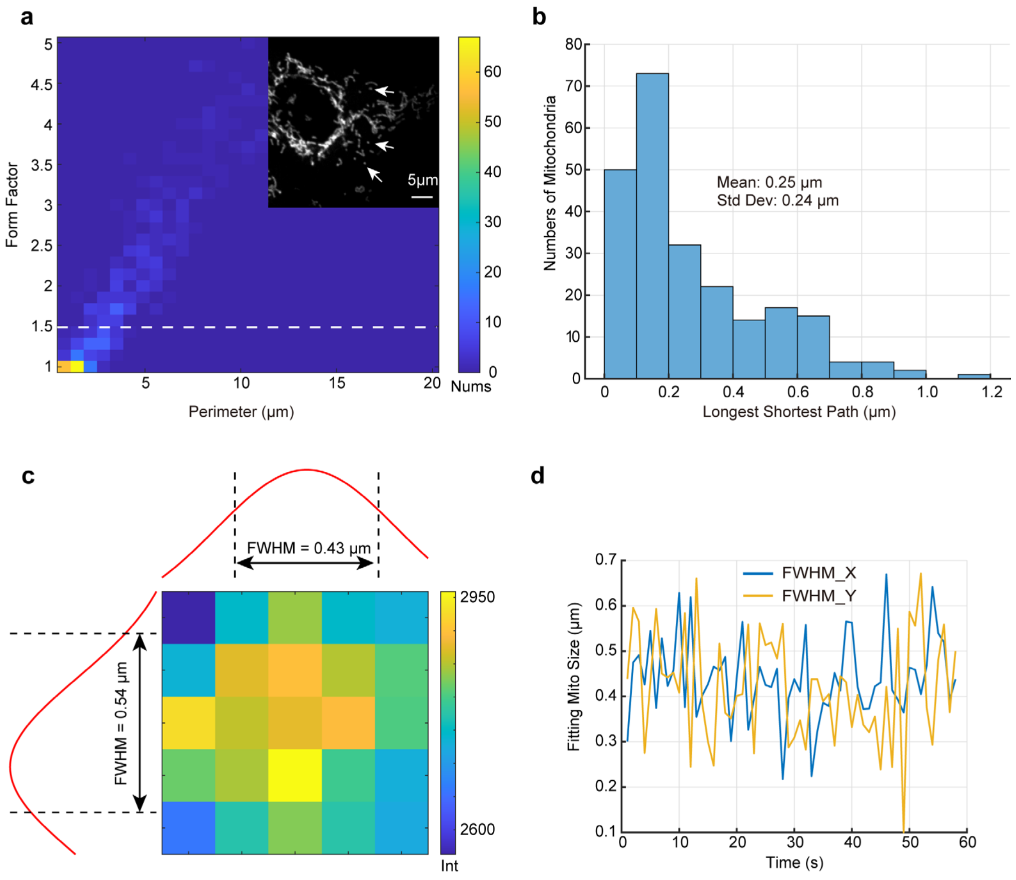

Supplementary Figure 14. Mitochondrial size analysis during mitophagy. **(a)** The heatmap of perimeters of mitophagy versus form factor, where form factor indicates the mitochondrial shape (the values close to 1 indicate a nearly circular morphology). **(b)** The histogram distribution of mitochondrial longest shortest path. Only the form factor lower than 1.5 were analyzed. The longest shortest path approximates the diameter of mitochondrial. **(c)** The 2D image of the tracked mitochondrion in Fig. 3 by mapping fluorescence photons to the laser focus positions at their corresponding time points. Bin time = 1 s. (**d**) The full width at half maximum (FWHM) of the mitochondrial image in (**c**) as a function of time during tracking.

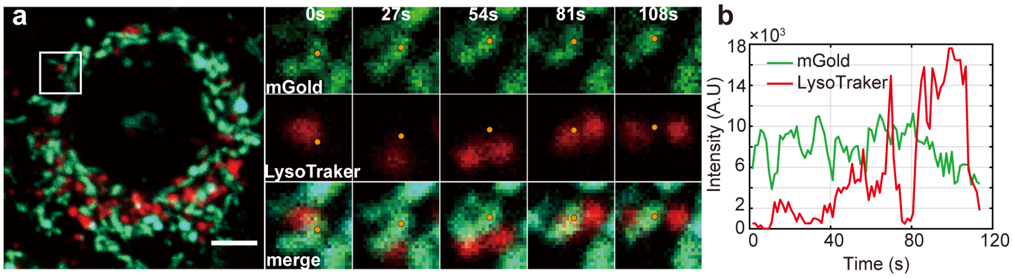

Supplementary Figure 15. Monitor the mitophagy process with confocal microscopy. (**a**) Time-lapse confocal imaging of mitochondria and lysosome during mitophagy process, with mitochondria show in green and lysosomes show in red. Scale bar = 5 μm. **(b)** The fluorescence intensity of mGold on mitochondria and lysosome as a function of time in positions marked with orange dot in **(a)**. The mitochondria were labeled with mGold and the lysosomes were labeled with LysoTracker Deep Red. The orange dot positions were determined by finding the largest fluorescence intensity pixel in each mitochondrion image. The intensity of mGold was decreased when mitochondria merged with lysosome.

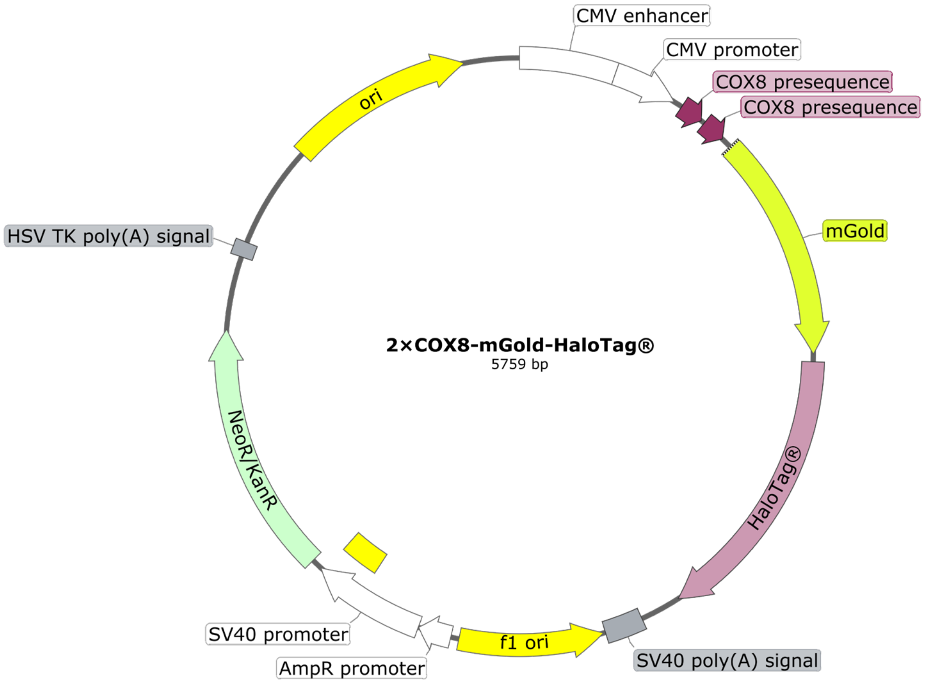

Supplementary Figure 16. Domain structure of 2×COX8-mGold-HaloTag used in mitophagy experiments.

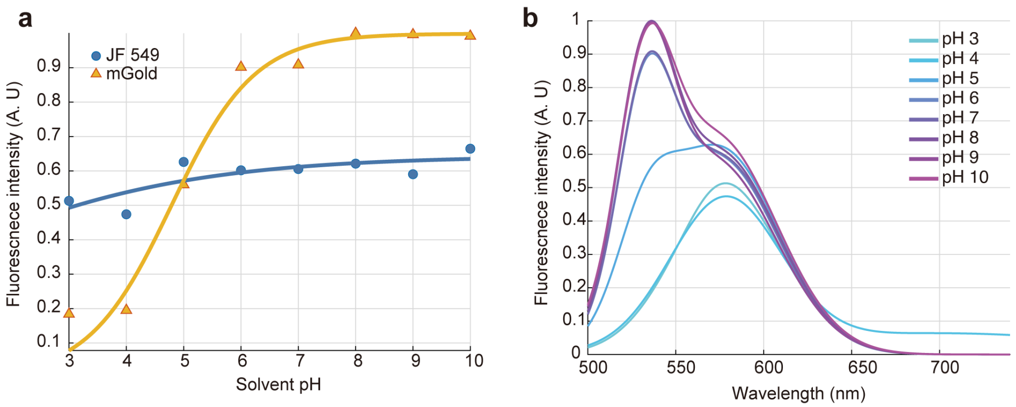

Supplementary Figure 17. pH titration of mGold and JF549 dye. (**a**) The fluorescence intensity of mGold (yellow triangle) and JF 549 (blue dot) as a function of pH of solvent. **(b)** The fluorescence spectral profile of mGold-JF549 in solutions with various pHs. Due to the differences in the absorption spectra of mGold and JF549, the combined spectrum in various pH buffers[1] was synthesized under 488 nm and 540 nm laser excitation, respectively. Specifically, after measuring the spectra of the mGold-JF549 mixture under 488 nm and 540 nm laser excitation separately, the spectra shown in (**b**) were obtained by summation and normalization.

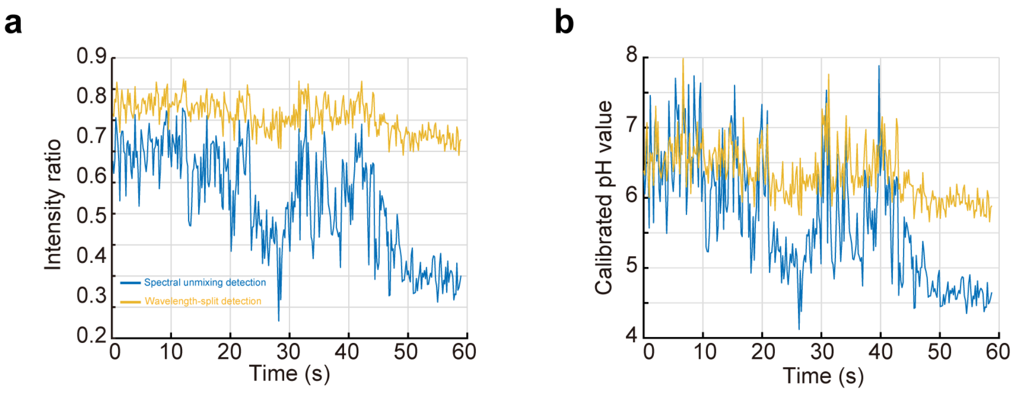

Supplementary Figure 18. Comparison of the 3D-SpecDIM-enabled spectral unmixing ratiometric fluorescence detection and the conventional dual-channel ratiometric detection. (**a**) The intensity ratio comparison of dual-channel ratiometric detection (yellow line) and 3D-SpecDIM-enabled spectral unmixing detection (blue line). **(b)** Similar comparison as in (**a**), with the corresponding mGold/JF549 intensity ratio converted to a calibrated pH. The pH range is scaled to start at 6.5.

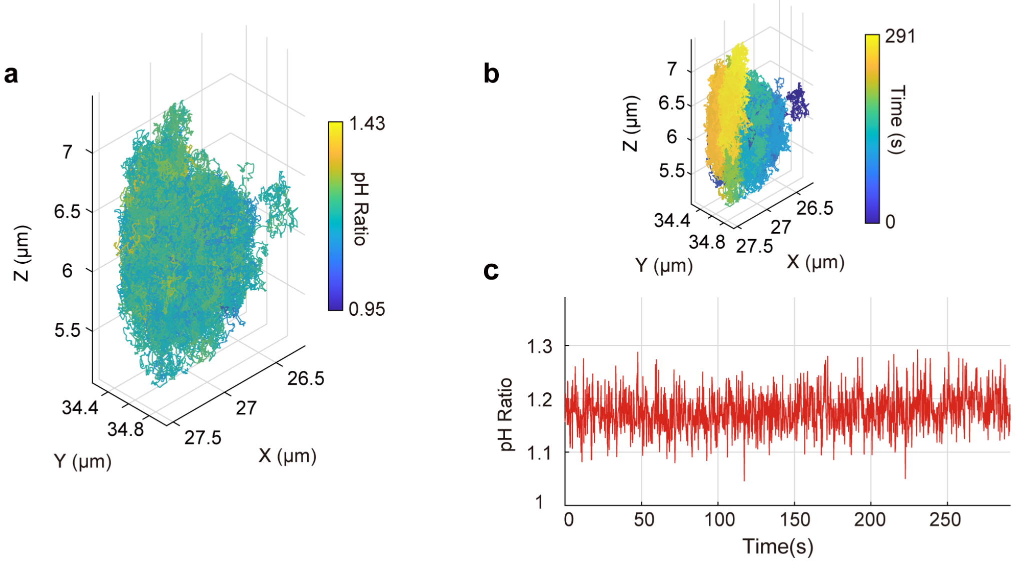

Supplementary Figure 19. Spectrum dynamics tracking of pH-sensitive probe labeled mitochondrion in cells without mitophagy. (**a**, **b**) 3D trajectory of a mitochondrion in live cell. The color indicates pH ratio (**a**) or time (**b**). (**c**) pH ratio as a function of time. The excitation laser power of 488 nm, 561 nm, and 638 nm are the same with experiment in **Figure 3f** in main text.

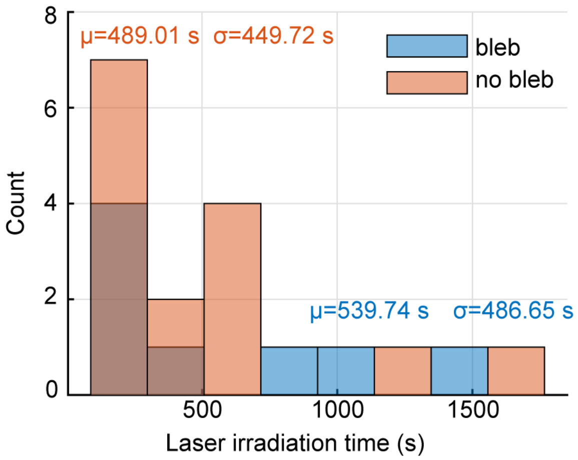

Supplementary Figure 20. The histogram distribution of laser irradiation time for blebbing (blue, n = 15 trajectories) and no blebbing (orange, n = 8 trajectories) events. The mean irradiation times of μ=539.74 s (σ=486.65 s) for blebbing events and μ=489.01 s (σ=449.72 s) for no blebbing events.

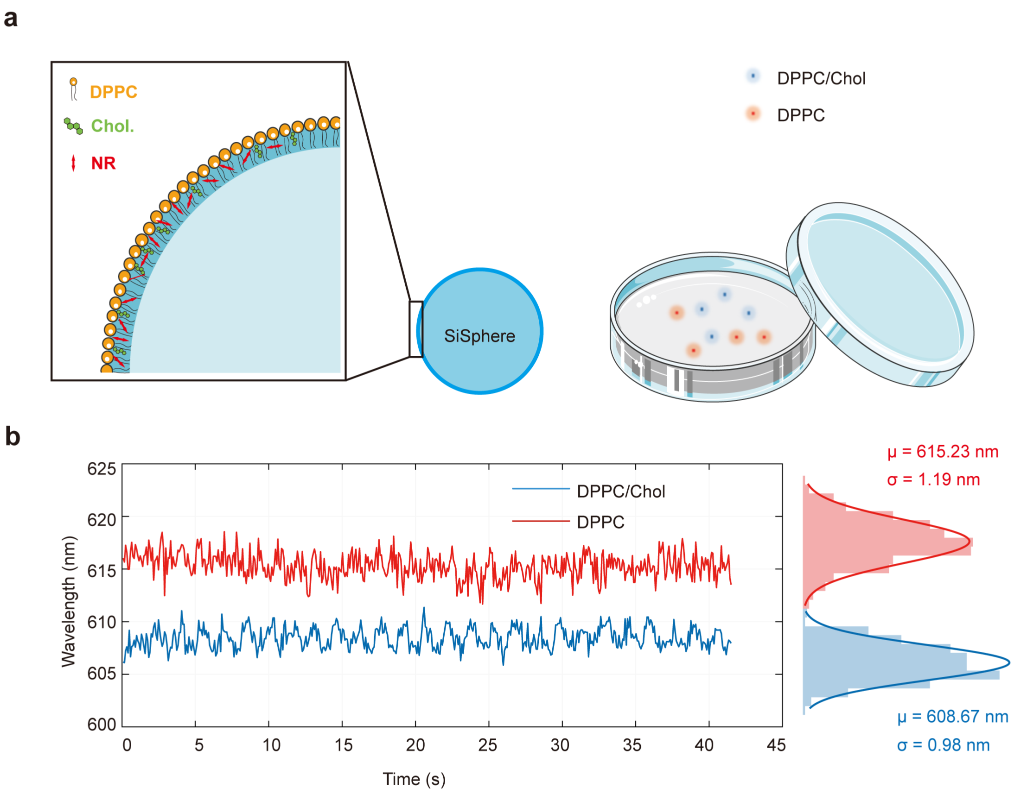

Supplementary Figure 21. **Characterize the polarity of lipids membrane with 3D-SpecDIM**. (**a**) Schematic diagram of the structure of SLB-coated 100 nm silicon spheres, where DPPC represents 1,2-dipalmitoyl-sn-glycero-3-phosphocholine, Chol represents cholesterol, and NR represents Nile Red. **(b)** With the existence of cholesterol, the polarity of lipids membrane was decreased, and the fluorescence spectrum shows blue shift.

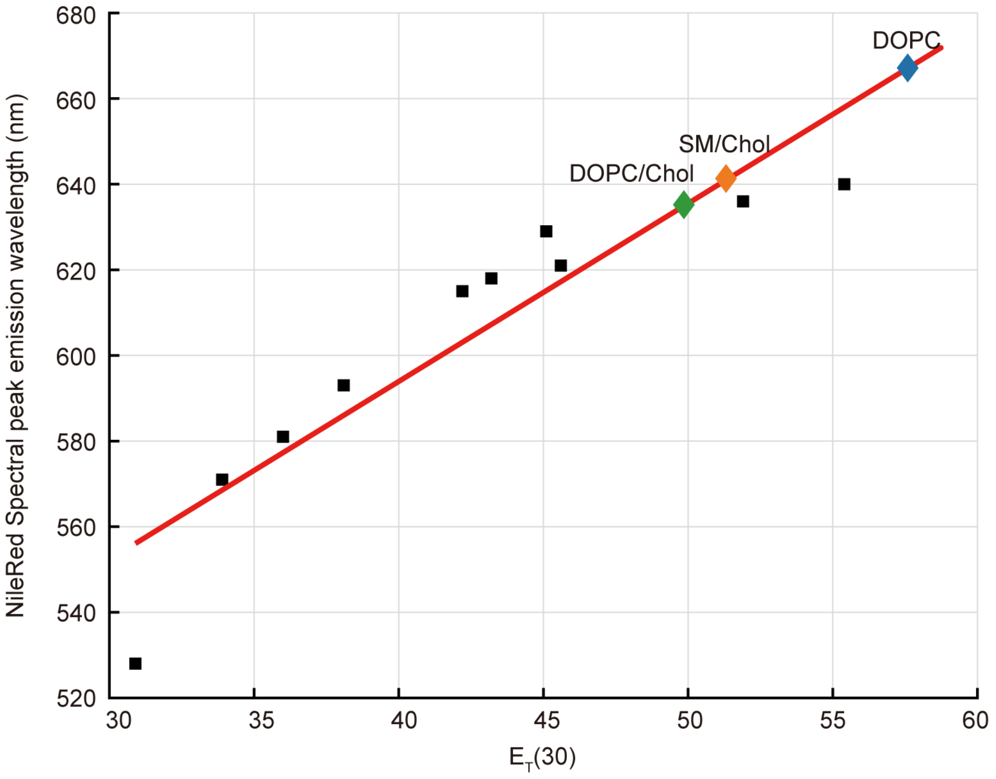

Supplementary Figure 22. The relationship between spectral peak emission wavelength of Nile Red and $E_T(30)$, a commonly used parameter for quantifying the polarity. The black square dots indicate the Nile Red spectral peak emission wavelength position in different solution with corresponding $E_T(30)$, a parameter used for characterizing the polarity of solvent. The green diamond dot, orange diamond dot and blue diamond dot show the Nile Red spectral peak emission wavelength position in different solutions as marked on the plot figure. Therefore, the polarity of these solution, or the value of their $E_T(30)$, can be extracted.

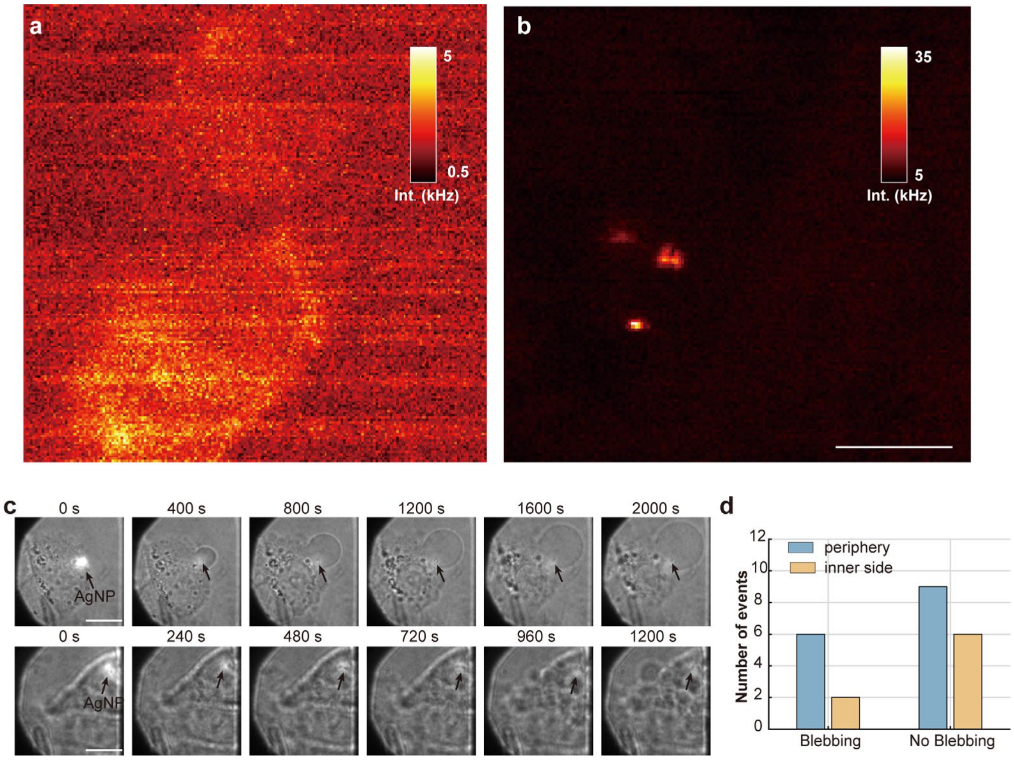

Supplementary Figure 23. Signal to background ratio characterization of AgNPs scattering imaging in cell and the bright field images of cellular blebbing process. **(a)** Scanning image of Hela cells without AgNPs. Hela cells were labeled with Nile Red. The 488 nm laser was used for excitation and a 488/10 nm bandpass filter coupled with a polarizer were used in the detection path. **(b)** Scanning image of Hela cells with AgNPs. **(c)** Time-lapse bright field images of cellular blebbing imaging process. The upper panel shows cellular blebbing occurred at the periphery of cell, while the lower panel shows cellular blebbing occurred on the inner side of cell. Scale bar: 10 µm. (**d**) Blebbing occurred frequency at different cell position (n = 24 trajectories). We examined the blebbing positions occurred on cell. The results show that blebbing occurred more frequently at the cell periphery (43.75%) than on the inner side (25%).

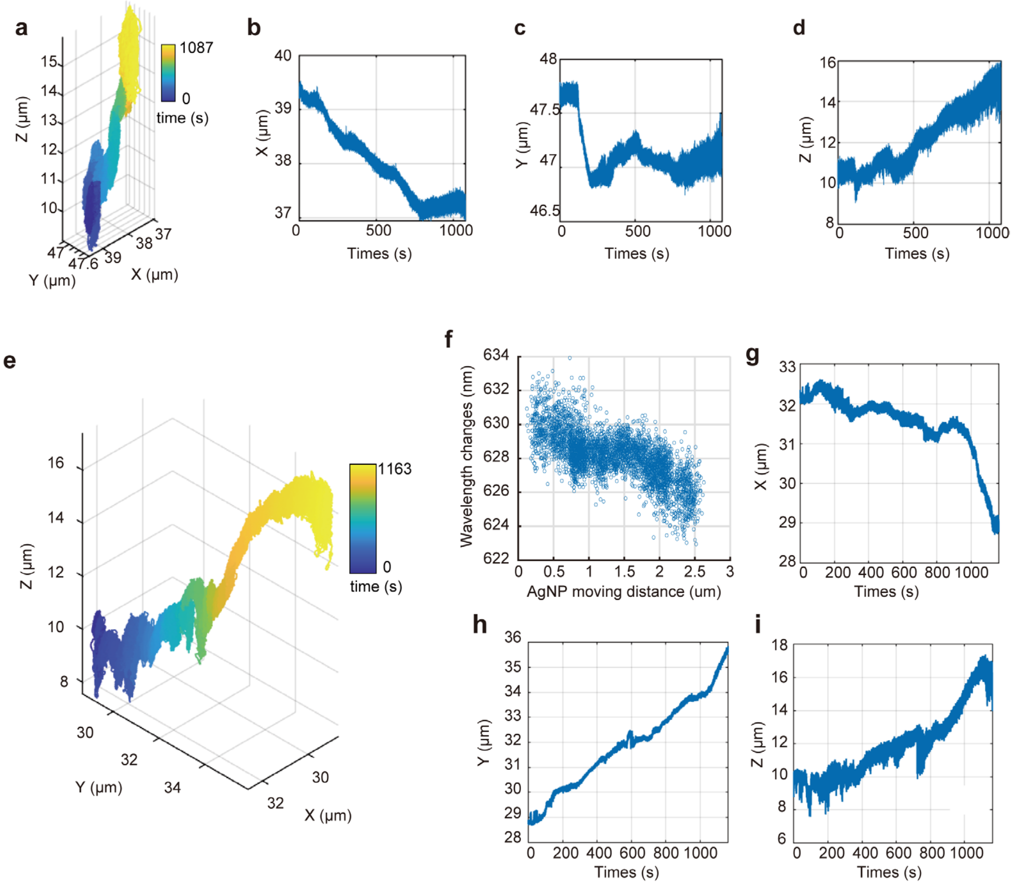

Supplementary Figure 24. 3D trajectories of AgNPs during celluar blebbing. (a) 3D trajectory of AgNP during blebbing. The AgNP initially located on the inner side of cell. (**b-d**) x, y, and z position of AgNP as a function of time. **(e)** 3D trajectory of AgNP during blebbing. The AgNP initially located at the periphery of cell. (**f**) Fluoruescnece spectrum peak emission wavelength changes of Nile Red as a function of AgNP moving distance. (**f-h**) x, y, and z position of AgNP as a function of time.

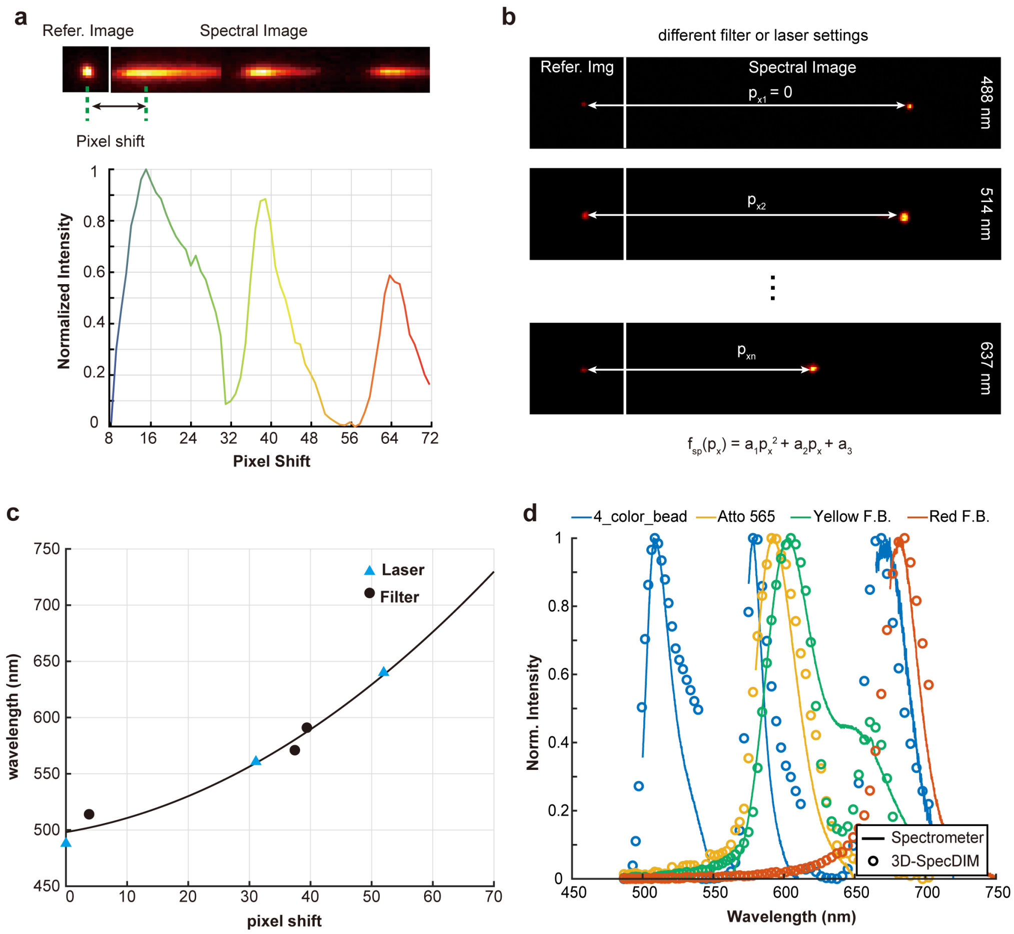

Supplementary Figure 25. Spectral registration. (a) Top panel: the reference image (left) and spectral image (right) of four-colors fluorescent bead (Thermo Fisher Scientific, TetraSpeck™, T7279) on EMCCD. The spectral image was generated by horizontally flipping the raw spectral image. Bottom panel: the fluorescence intensity distribution versus pixel shift for spectral image. **(b)** Spectral calibration with different narrow bandpass filters and lasers. **(c)** Convert the pixel shift between the reference image and the spectral image into spectral wavelengths using quadratic polynomial fitting. **(d)** Comparison of spectra acquired with 3D-SpecDIM and a commercial spectrofluorometer. Four different fluorophores were analyzed: four-color fluorescent bead (Thermo Fisher Scientific, TetraSpeck™, T7279, blue line and circle), Atto 565 dye (yellow line and circle), yellow fluorescent bead (FSSY002, Bangs Lab, green

line and circle), and red fluorescent bead (FSFR002, Bangs Lab, red line and circle). The root mean square error (RMSE) between the spectral data from the two systems was calculated to be 0.23.

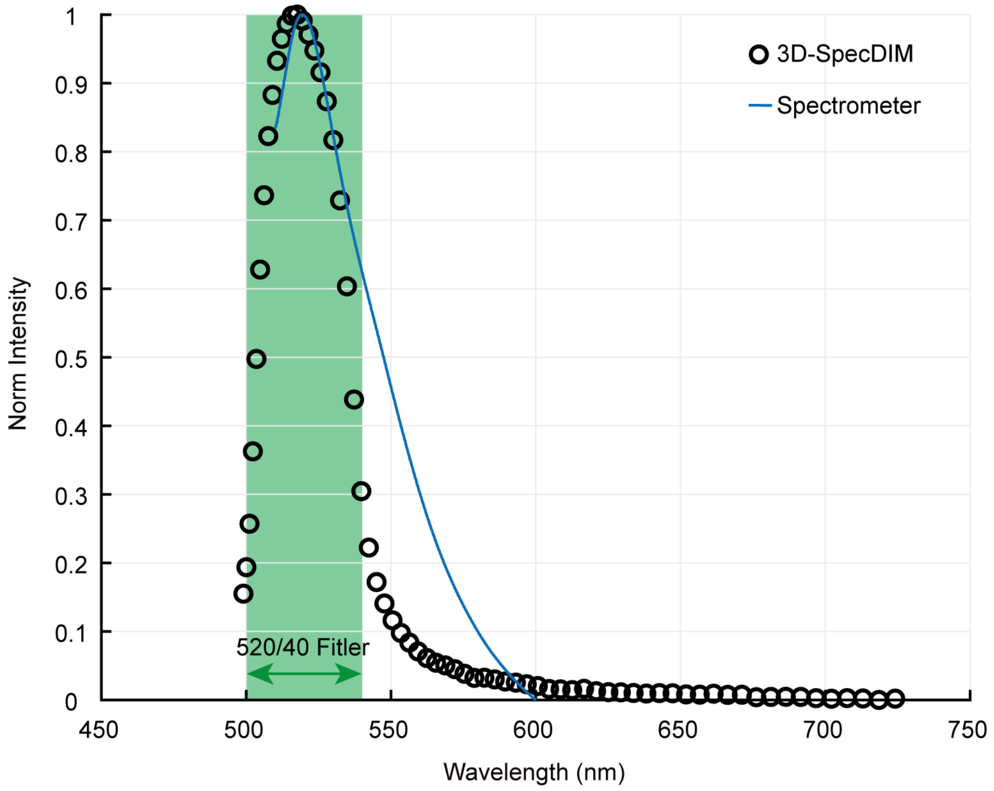

Supplementary Figure 26. Comparison of green fluorescent dead spectra acquired using 3D-SpecDIM (circle) and a commercial spectrometer (solid line).

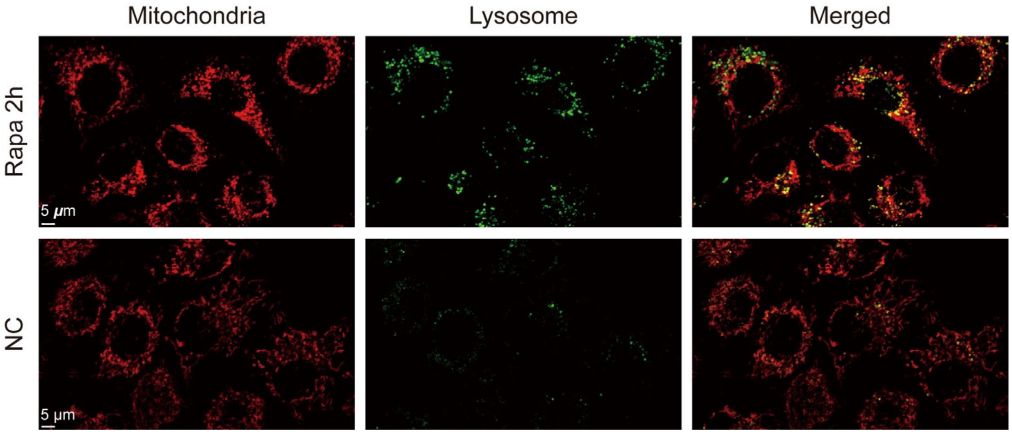

Supplementary Figure 27. Confocal images of mitochondria and lysosome during mitophagy. Upper panel: with rapamycin; lower panel: without rapamycin. The mitochondria were labeled with mGold-HaloTag-JF549, excited by 488 nm laser and the lysosomes were labeled with LysoTracker Deep Red, excited by 640 nm laser.

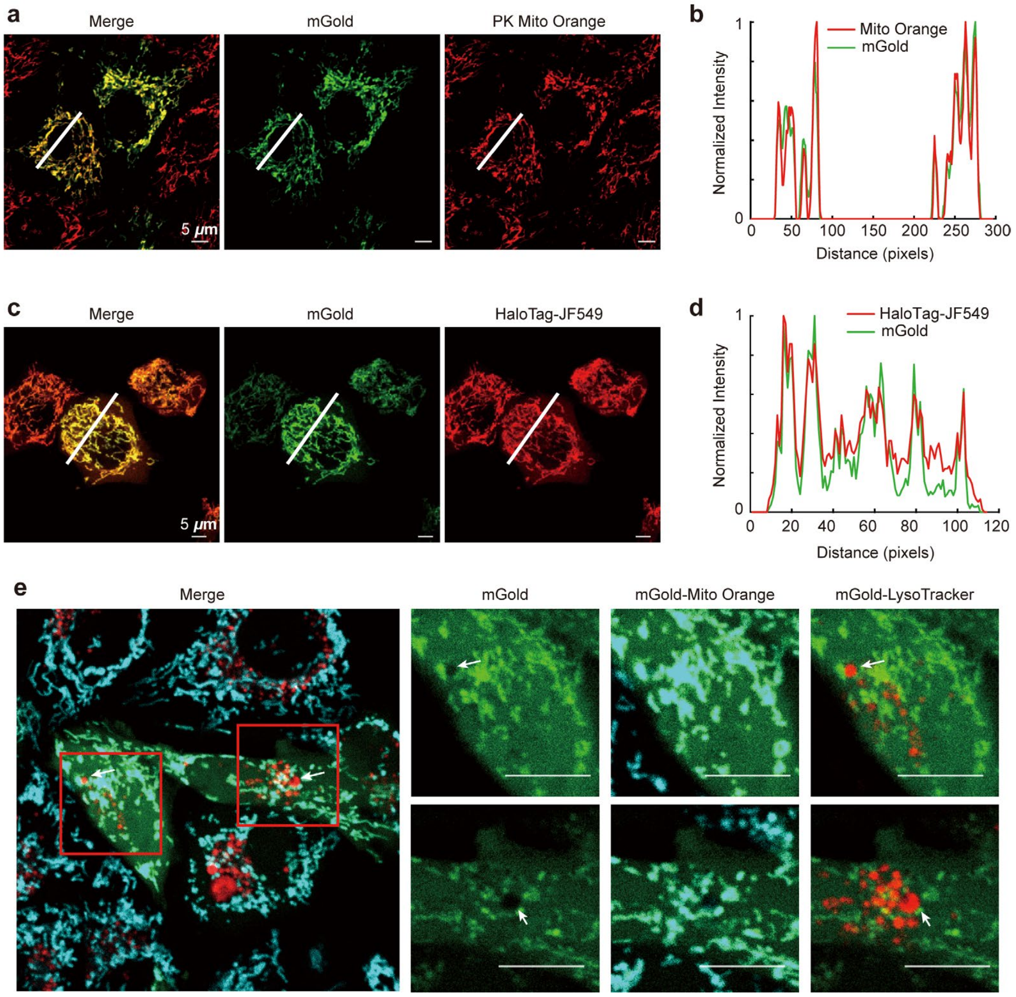

Supplementary Figure 28. Comparison between 2×cox8-mGold-HaloTag and mitochondrial dyes. (a) Confocal images of Hela cells stained with 2×cox8-mGold and commercial mitochondrial dyes, PK Mito Orange (PKMO-1, genvivotech). **(b)** Overlayed intensity distribution profile of mGold and PK Mito Orange along the white line shown in **(a)**. **(c)** Confocal images of Hela cells stained with 2×cox8-mGold-HaloTag and HaloTag dyes JF549. **(d)** Overlayed intensity distribution profile of mGold and JF549 Orange along the white line shown in **(c)**. **(e)** Confocal images of Hela cells stained with 2×cox8-mGold, PK Mito Orange, and LysoTracker Deep Red. Scale bar: 10 μm.

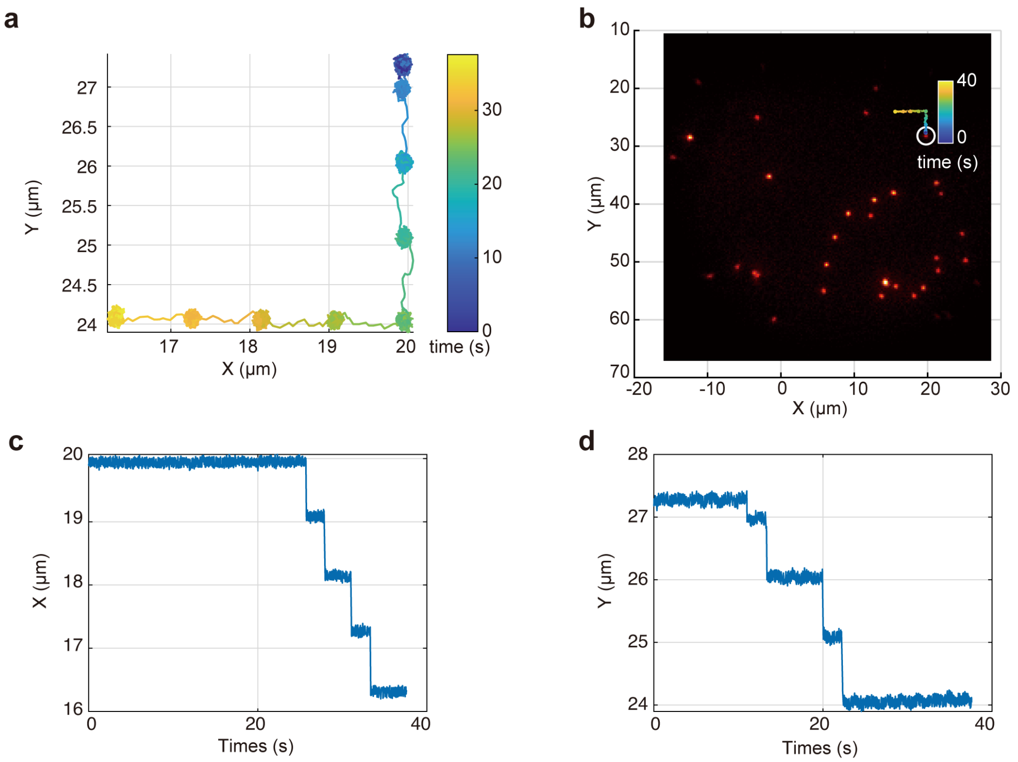

Supplementary Figure 29. Align the tracking trajectory position with EMCCD image. (a) Trajectories of a fluorescent bead with its position controlled by a displacement stage. **(b)** Register trajectory on EMCCD image. The white circle in the image shows the position of tracking point in the EMCCD recorded image. **(c, d)** x and y position as a function of time.

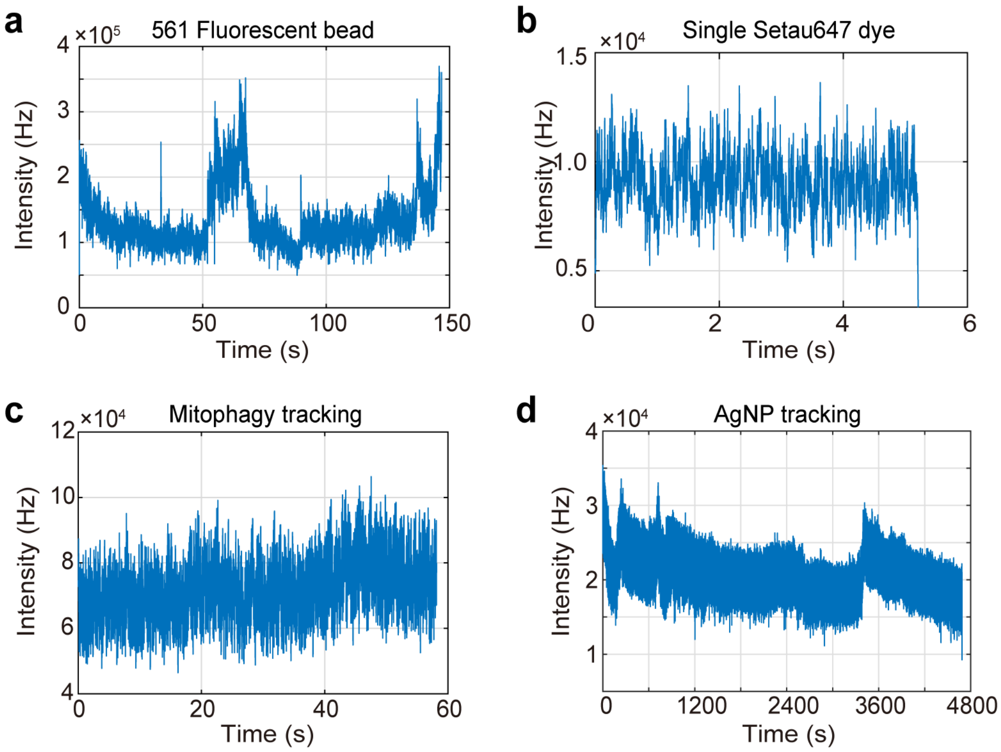

Supplementary Figure 30. The APD signal as a function of time for Fig. 2a (a), Fig. 2 g-h (b), Fig. 3b-c (c) and Fig. 4b. It is worth noting that the intensity fluctuations should be attributed to changes in the photon collection efficiency of the objective lens caused by variations in axial position.

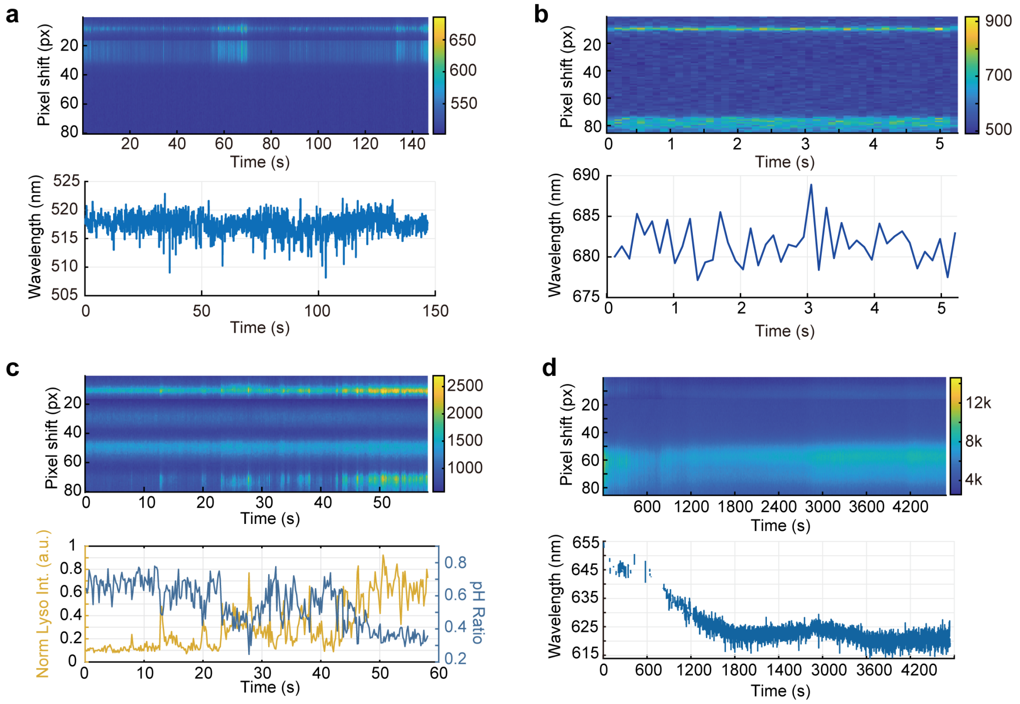

Supplementary Figure 31. Kymograms for the relevant trajectories in main text. The top panel corresponds to the kymograms of the spectral images, in which the the upper bright line shows the reference position signal while the lower wider line shows the spectral signal. The bottom panel in (a), (b), and (d) show the spectral peak emission wavelength position as a function of time. The bottom panel in (c) is the same with Fig. 3f. (a) Kymogram for trajectory Fig. 2a-c. (b) Kymogram for trajectory Fig. 2g-i. (c) Kymogram for trajectory Fig. 3b-f. (d) Kymogram for trajectory Fig. 4b-e. Only successfully fitted spectral data points are presented. At some time points, the signal-to-noise ratio was too low to obtain a valid spectral peak emission wavelength estimation.

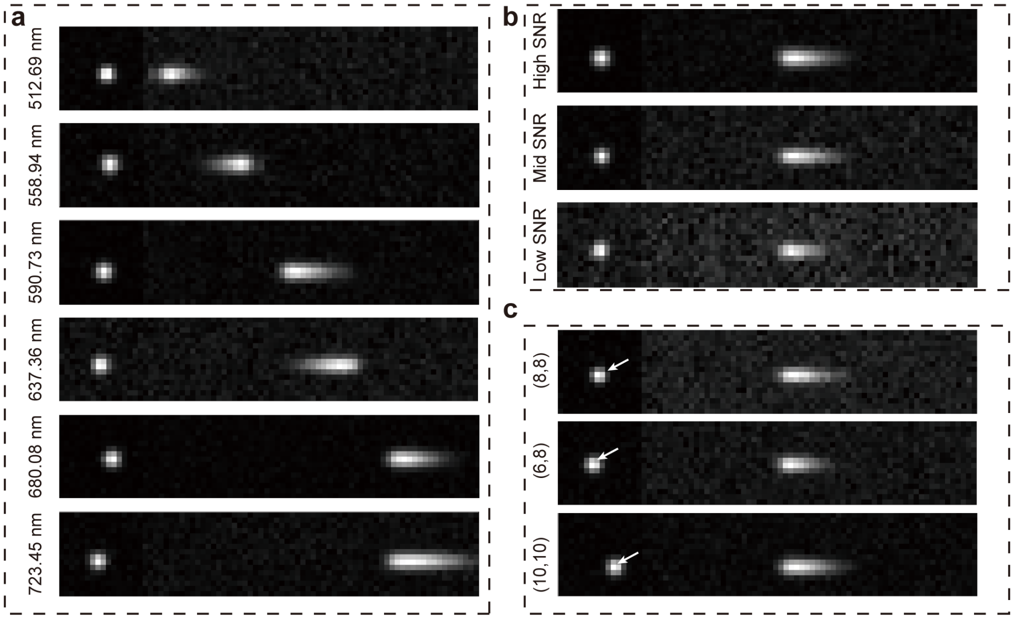

Supplementary Figure 32. Simulated datasets for ViT model training. (a) Simulated datasets with different spectral peak emission wavelength from 510 nm to 740 nm. **(b)** Simulated datasets with different signal-to-noise ratio levels. **(c)** Simulated datasets with the particles located at different positions on image.

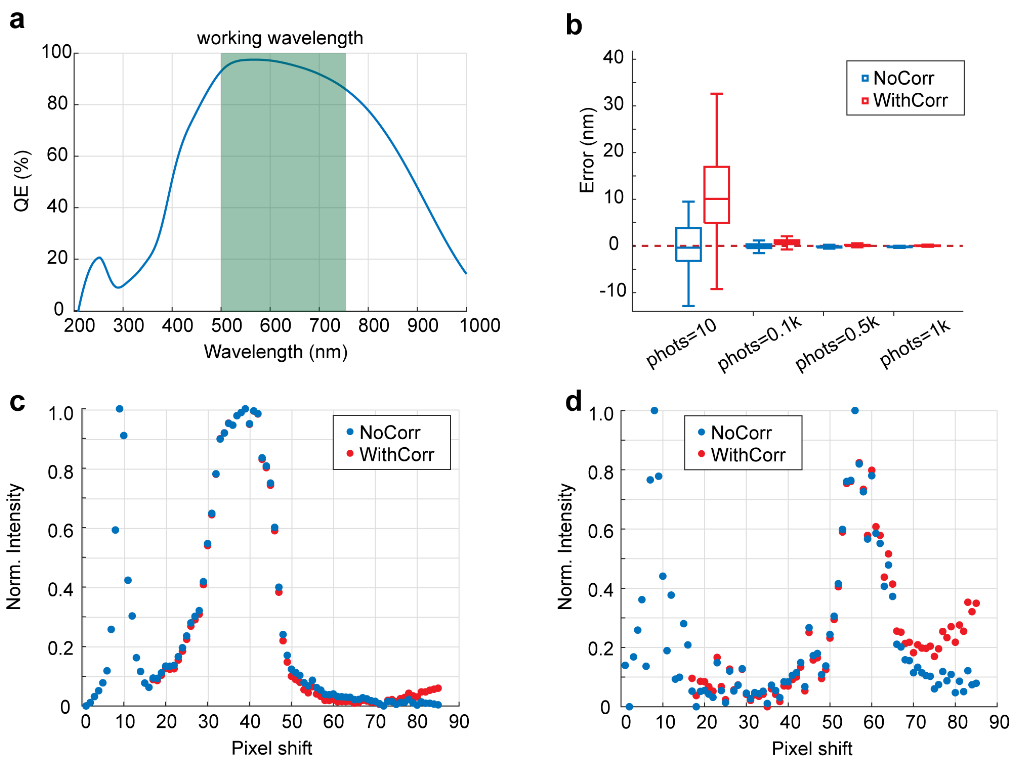

Supplementary Figure 33. Evaluation of QE correction in spectral peak emission wavelength estimation. (a) Quantum efficiency (QE) curve of the EMCCD sensor. The shaded region (500–740 nm) indicates the working spectral range of the 3D-SpecDIM system. **(b)** Monte Carlo simulation of spectral peak emission wavelength estimation under different photon counts. Blue boxes: no QE correction; red boxes: with QE correction. QE correction leads to increased error under low-photon conditions (e.g., 10 photons and 10 background), while the difference becomes negligible at high photon counts (≥500). **(c)** Comparison of spectral profiles with and without QE correction for a high-intensity fluorescent bead (corresponding to Fig. 2e, emission rate > 5 MHz). The peak emission wavelength difference is minimal, demonstrating that QE correction has limited impact under high signal levels. **(d)** Comparison of spectral profiles for a single Atto565 molecule with and without QE

correction. The QE-corrected spectrum shows amplified noise, consistent with the simulation results.

Supplementary Tables

Supplementary Table 1. Temporal resolution analysis of 3D-SpecDIM system.

EMCCD parameters		*Array size of EMCCD*		
		512×512	25×275	275×25
	Exposure time	**Frame rate**		
3.3 µs vertical clock speed 10 MHz pixel readout speed	10 *µ*s	28 FPS	45 FPS	141 FPS
	5 ms	24 FPS	37 FPS	83 FPS
	100 ms	7.3 FPS	8.2 FPS	9.3 FPS
0.3 µs vertical clock speed 17 MHz pixel readout speed	10 *µ*s	54 FPS	97 FPS	644 FPS
	5 ms	43FPS	67 FPS	154 FPS
	100 ms	8.5 FPS	9.1 FPS	9.8 FPS

Supplementary Table 2. Cell blebbing occurrence analysis under different conditions.

Experiments setup			Observed blebbing?
Laser power (measured after objective lens)	Dyes concentration	AgNPs concentration	
488 nm: 6.9 µW<br>561 nm: 0.3 - 2 µW	1 µM NileRed for 20 min incubation	10 µg/mL for 10 min incubation	yes
488 nm: 3.5 µW	1 µM NileRed for 20 min incubation	-	yes
488 nm: 0.5 µW	1 µM NileRed for 20 min incubation	-	no
488 nm: 6.9 µW	-	10 µg/mL for 10 min incubation	no
488 nm: 6.9 µW	1 µM NileRed for 20 min incubation	-	yes
488 nm: 6.9 µW	100 nM NileRed for 20 min incubation	-	no
488 nm: 6.9 µW	-	10 µg/mL for 10 min incubation	no
488 nm: 6.9 µW	1 µM Cellmask for 20 min incubation	10 µg/mL for 10 min incubation	no
488 nm: 6.9 µW	1 µM DiD for 20 min incubation	10 µg/mL for 10 min incubation	no

Supplementray Tables 3. Statistics data of fluorescence spectral peak emission wavelength changes during cell blebbing.

	Number of tracks	Average spectral changes
Blebbing at the periphery of cell	5	-7.66 ± 3.87 nm
Blebbing on the inner side of cell	2	0.03 ± 0.24 nm
Without blebbing at the periphery of cell	4	-2.49 ± 1.30 nm
Without blebbing on the inner side of cell	6	-0.36 ± 1.87 nm

Supplementary Table 4. Optical path configurations and experimental parameters.

Experiments	Laser and Power (measured after objective lens)	F1 filter	F2 filter	F3 filter	Exposure time used on EMCCD
Fluorescent beads spectral tracking	Green beads, 488 nm: 0.1 μW	520/40 (ET520/40m, Chroma)	-	-	10 ms
	Yellow beads, 561 nm: 0.06 μW	630/92(FF01-630/92, Semrock)	-	-	10 ms
Single fluorescent molecule spectral tracking	Setau647N 638 nm: 2.0 μW	706/95 (ET706/95m, Chroma)	-	-	100 ms or 150 ms (Fig2g-i)
	Atto 647N 638 nm: 2.0 μW	706/95 (ET706/95m, Chroma)	-	-	100 ms
	Atto565N 561 nm: 2.0 μW	600/52(FF01-600/52, Semrock)	-	-	100 ms
	BSA-Atto565N 561 nm: 1.5 μW	600/52(FF01-600/52, Semrock)	-	-	-

Mitophagy imaging	488 nm: 0.05-0.15 μW<br>561 nm: 0.1 μW	488/561 (ZET488/561m, Chroma) or 405/488/561/638 (ZT405/488/561/640mv2, Chroma)	-	630/92 or 600/52(FF01-600/52, Semrock)	30 ms
Cellular membrane blebbing imaging	488 nm: 6.9 μW<br>561 nm: 2.0 μW	-	Polarizer (GLP10-A, Lbtek) and 488/10 (FF01-488/10, Semrock)	630/92(FF01-630/92, Semrock)	30 ms

Supplementary Table 5. Preparation of Silicon spheres coated with SLB.

Volume of 40 μg/mL sphere solution(μL)	Volume of Tris Ca2+ buffer (μL)	Volume of lipid SUV solution (μL)	Centrifugation speed (rpm)
1.4	53.6	125.0	2000

Supplementary Table 6. Comparison of measured spectral peak emission wavelength values between 3D-SpecDIM and Commerical spectrometer.

	atto565N	SY540	FR640	Tdeepred	TGreen	Torange
3D-SpecDIM (nm)	593.0	604.8	685.3	668.8	508.6	578.0
Commercial Spectrometer (nm)	592.2	603.1	680.7	674.8	507.7	577.7
Error (nm)	+0.8	+1.7	+4.6	-6.0	+0.9	+0.3

Supplementary Table 7. Spectral detection performance comparison between 3D-SpecDIM and Zeiss LSM 980 spectral scanning confocal microscope.

	Spectral scanning step size/precision	Scanning area (pixels)	Time resolution	Measuring dimensions
3D-SpecDIM	~0.5 nm	-	~0.1 s	3D
Zeiss-Commercial Microscope LSM980	3 nm (from 568 nm to 740 nm)	16.1×16.1 μm^2 (64×64 pixels)	117 s	2D
		84.9×84.9 μm^2 (1024x1024 pixels)	634 s	2D
	5 nm (from 568 nm to 740 nm)	16.1×16.1 μm^2 (64×64 pixels)	46 s	2D
		84.9×84.9 μm^2 (1024×1024 pixels)	202 s	2D

Supplementary Note

Supplementary Note 1: Silicon spheres coated with supported lipid bilayers.

Small unilamellar vesicles (SUVs) containing different components of lipids were prepared using sonication. Specifically, a solution mixed with 35.24 μL (25 mg/mL in chloroform) of 1,2-dipalmitoyl-sn-glycero-3-phosphocholine (DPPC, MCE; 63-89-8), and 31.28 μL (10 mg/mL in chloroform)) of cholesterol (Chol, Aladdin; C104028) or 58.4 μL DPPC only was dried under nitrogen and left in vacuum for 12 hours to completely evaporate the chloroform. Then 2 mL of Tris Ca^{2+} buffer (100mM NaCl, 3mM Ca^{2+}, 10mM Tris, pH 7.4) was added to the dry lipid mixture and hydrated in a 55°C water bath for one hour, forming a vesicle suspension. This vesicle suspension was then put to a 4°C refrigerator for 4 hours to achieve a final concentration of 1 mM lipid mixture. The suspension was sonicated with a probe-type ultrasonicator at a constant amplitude of 60% (maximum power of 130W) for 10-15 minutes, pausing for 10 seconds after every 30-40 seconds of sonication in an ice bath to form an SUV suspension. Then, 2 mL of SUV suspension was transferred into two sterile 1.5 mL centrifuge tubes in 900 μL aliquots each and sealed with sealing film. The tubes were centrifuged at 15,000g for 1 hour at 4°C, leading to the appearance of a black substance at the bottom of the tubes (probe fragments and unbroken vesicles). For each tube, 800 μL of supernatant was collected into 1.5 mL centrifuge tubes and sealed, then stored in a 4°C refrigerator. A 100 μL aliquot of the SUV solution was then taken for particle size measurement with dynamic light scattering, resulting in 1.6 mL of 1 mM DPPC/chol or DPPC SLB solution.

The 150-nm silicon spheres (Xfnano, 7440-21-3) were diluted with Tris Ca^{2+} to 40 μg/mL. According to the **Supplementary Table 5**, the spheres were further diluted to a certain concentration. The diluted microsphere solution was

then heated in a 65°C water bath before being mixed with the lipid SUV solution. The mixture was maintained in the water bath for 30 minutes, with rotation every 5 minutes, and then gradually cooled to room temperature. Following the speeds indicated in the table, the mixture was centrifuged for 5 minutes. After centrifugation, the Tris Ca^{2+} supernatant was replaced with Tris buffer (100 mM NaCl, 10 mM Tris, pH 7.4), resulting in 180 μL of SLB-wrapped silicon sphere solution.

For Nile Red staining, 1 μL of a 3 mM Nile Red stock solution was dissolved in 3 mL of Tris buffer to make a 1 μM Nile Red solution. Then, 5.76 μL of 1μM Nile Red was added to the SLB-wrapped silicon sphere solution (final concentration of 32 nM) and incubated in the dark at room temperature for 10 minutes.

Supplementary Note 2: Vision Transformer and domain adaption-based spectral feature recognition for improving spectral imaging precision.

Vision Transformer (ViT) Network for Spectral Feature Recognition. To improve spectral detection accuracy, we developed a spectral feature recognition method based on the Vision Transformer (ViT) model[2]. The input to the ViT model is the spectral image, which has dimensions of 16×80 pixels (**Supplementary Figure. 2**). In this image, the left 16×16 region contains reference fluorescence positioning information, while the right 16×64 region represents the dispersive spectrum, where the pixel shift corresponds to emission wavelengths. The ViT processes these spectral images by initially dividing the input image I into a series of N patches I_p, with each patch reshaped into a vector of dimension $P^2 \cdot C$, where P is the patch size and C is the number of color channels. These vectors are projected into a higher-dimensional space D using a matrix $E \in \mathbb{R}^{(P^2 \cdot C)\times D}$, forming a sequence of patch embeddings. Learnable positional embeddings are then incorporated into the patch sequence to retain spatial information, forming the input to the transformer encoder. Following the encoding process, the output is passed through a single-layer multilayer perceptron (MLP), which generates the spectral peak emission wavelength—a single numerical value representing the center of the spectral distribution for the analyzed fluorescence spectrum. This workflow ensures precise spectral feature extraction and robust peak emission wavelength detection from spectral images. The input to transformer encoder can be represented as:

$$X_{patch} = \left[I_{p_1}E, I_{p_2}E, \dots, I_{p_N}E\right] + E_{\mathrm{pos}}, \qquad (1)$$

The encoder consists of multiple layers, each comprising two main components: Multi-Head Self-Attention (MHSA) and Feed-Forward Networks (FFNs). MHSA is computed as:

$$Attention(Q, K, V) = softmax\left(\frac{QK^T}{\sqrt{d_k}}\right)V, \quad (2)$$

where Q, K, and V are queries, keys, and values obtained by projecting the input patch X_{Patch}, and d_k is the dimension of the key, ensuring proper scaling. This attention mechanism allows ViT to focus on relevant parts of the image by assigning importance weights to different patches. Following MHSA, each encoder layer applies position-wise FFNs consisting of two linear transformations with a ReLU activation. Specifically, for an input vector x, the FFN is:

$$FFN(x) = \max(0, xW_1 + b_1)W_2 + b_2 \quad (3)$$

Where W_1, W_2, b_1, and b_2 are learnable parameters. Layer normalization and residual connections are also employed around both MHSA and FFN modules, enhancing training stability and allowing deeper models.

By processing images through these sequential transformer layers $x' = LayerNorm\big(x + Sublayer(x)\big)$, ViT captures complex patterns and dependencies among image patches, leveraging the power of the transformer architecture for spectral peak emission wavelength recognition tasks. This design enables the extraction of rich feature representations and explicitly makes the model focus on spatial relationships within the image, marking a superior outcome compared to convolutional networks.

Domain adaption strategy. While the mentioned model demonstrates impressive performance on simulated datasets, practical applications often

reveal that simulated training data (source domain) may not encompass the full range of variability encountered in the target domain. To address this challenge, we implemented a novel approach, the Regression Margin Disparity Discrepancy (RMDD), to evaluate and reduce distribution discrepancies in domain adaptation tasks (**Supplementary Figure. 5b**)[3]. In short, RMDD is a measure of the difference in distribution between the source domain and the target domain, with a particular focus on the difference in the context of margin loss. Central to this approach is the utilization of a Gradient Reversal Layer (GRL) that strategically modifies the direction of gradient flow during backpropagation, thereby encouraging the model to learn features that are invariant across domains. During the forward pass, the GRL acts as an identity function, allowing data to pass through unchanged. However, during the backward pass, it multiplies the gradient by a negative constant ($-\lambda$). This operation effectively reverses the direction of the gradient flow for the adversarial predictions, which is mathematically represented as:

$$GRL(\nabla_\theta L) = -\lambda \cdot \nabla_\theta L, \tag{3}$$

where $\nabla_\theta L$ denotes the gradient of the loss with respect to the model parameters θ, and λ is a hyperparameter that controls the strength of the gradient reversal. The RMDD loss function is defined as:

$$D_\gamma(\hat{y}_s, \hat{y}_{s_{adv}}, \hat{y}_t, \hat{y}_{t_{adv}}) = -m \cdot L_1(\hat{y}_s, \hat{y}_{s_{adv}}) + L_1(\hat{y}_t, \hat{y}_{t_{adv}}), \tag{4}$$

where D_γ represents the disparity between the actual predictions and their adversarial counterparts in the source $(\hat{y}_s, \hat{y}_{s_{adv}})$ and target domains$(\hat{y}_t, \hat{y}_{t_{adv}})$, and L_1 denotes the L1 loss. The term m is a pre-defined constant that scales the disparity in the source domain to control its influence on the overall adaptation process.

Loss function. The acquisition of loss function can be separated into two stages. For the training stage, it utilizes L1 loss to train on a simulated dataset. The loss function is calculated as follows:

$$L_{reg} = \frac{1}{N}\sum_{i} |y_i - \hat{y}_i|, \tag{5}$$

where N is the total number of observations in the dataset, y_i and $\hat{y}_i$ are the actual value and predicted value of the i-th observation, respectively. For the inference stage, we randomly mixed practical data with simulated data and used the average spectrum of fluorescent microspheres collected under the same conditions as pseudo-labels $y_{t_{pseudo}}$ for training. Thus, the overall loss during the domain adaptation stage is the weighted sum of regression loss and RMDD loss:

$$\begin{aligned} L_{domain} = L_{reg}(y_s, \hat{y}_s) + L_{reg}\left(y_{t_{pseudo}}, \hat{y}_t\right) \\ - D_\gamma(\hat{y}_s, \hat{y}_{s_{adv}}, \hat{y}_t, \hat{y}_{t_{adv}}), \end{aligned} \tag{6}$$

Training-data simulation. To generate spectral image sequences for training, we simulated the data in different imaging parameters, as illustrated in **Supplementary Figure. 32**. The parameters include background noise, spectral peak emission wavelength, diffusion coefficient, exposure time, photon count, and dichroic mirrors, et.al. Ultimately, we generated a total of 1,378,800 spectral images with varying signal-to-noise ratios, and the spectral peak emission wavelength ranging from 510 to 740 nm.

Integration of Spectral Feature Recognition Methods. To recognize spectral features in our experiments, we utilize both a conventional fitting-based approach and a deep learning-based approach. Illustrated in **Supplementary Figure. 5**, the composite image of 16×80 pixels includes a 16×16 segment for

molecular position and a 16×64 segment for spectral data after dispersion, with both segments undergoing normalization. Initially, the molecular coordinate p within the 16x16 segment is located. Aligning with the practical optical path configuration, we designate three spectral windows within the spectral image for 488 nm (from pixel 1 to pixel 22), 561 nm (from pixel 23 to pixel 43), and 640 nm (from pixel 44 to pixel 64) emissions. The channel with the largest signal is selected for normal distribution fitting to identify the pixel coordinates s of the spectral peak emission wavelength. The discrepancy in pixels between the spectral peak emission wavelength s and positional data p is calculated and converted into the actual wavelength of the spectral emission peak via a calibration function[4]. Alternatively, the neural network method inputs spliced images into a pre-trained model, which then outputs the spectral predictions directly.

Training procedure and Testing details. Totally 1,378,800 images across a range of signal-to-noise ratios were generated for the training of our deep learning model, reserving 10% of these for testing purposes. For domain adaptation, we collected tracks from fluorescent beads at 9 distinct signal-to-noise ratios to fine-tune the model. We further assessed the model's versatility by employing various spectral window settings. The training was conducted using two NVIDIA GeForce RTX 3090 graphics cards, with the programming carried out in the PyTorch-Lightning framework. Optimization was managed by the Adam optimizer, and we processed the data in batches of 128. Detailed information about our model is available on our GitHub page.

Supplementary Notes 3. Workflow and details of 3D-SpecDIM tracking and spectral data acquisition

1. Pixel-wavelength relationship calibration

Fixed four-color fluorescent beads (Thermo Fisher Scientific, TetraSpeck™, T7279) were placed on the microscope, and the 3D-SpecDIM system was utilized to track a fluorescent bead immobilized on the coverslip, capturing its spectral information using the EMCCD. The resulting image was stitched into a 16×80 matrix, with the 16×16 region representing the non-dispersed spatial channel, where the bead center was located at pixel coordinates (8, 8), and the remaining 16×64 region corresponding to the dispersed spectral channel for intensity analysis along pixel coordinates (**Supplementayr Fig. 25a**). Using different filters (514/30 nm, 577/20 nm, 591/6 nm) and measuring the spectral channel positions of lasers (490.2 nm, 561.7 nm, 636.8 nm), six paired datasets of pixel shifts and spectral wavelengths were obtained (**Supplementayr Fig 25b**). A quadratic polynomial fitting was then applied to derive the calibration function for converting pixel shifts into spectral wavelengths (**Supplementayr Fig 25c**), allowing accurate conversion of intensity variations along pixel shifts into the true wavelength distribution.

To demonstrate the accuracy of spectral detection, we compared the spectra acquired using the 3D-SpecDIM system with those obtained from a commercial spectrofluorometer (FS5, Edinburgh Instruments). For the commercial spectrometer measurements, emission spectra were recorded for four different fluorophores: four-color fluorescent beads (Thermo Fisher Scientific, TetraSpeck™, T7279), Atto 565 dyes, yellow fluorescent beads (FSSY002, Bangs Laboratories), and red fluorescent beads (FSFR002, Bangs Lab). As shown in **Supplementary Figure 25d**, the spectral data obtained with the 3D-SpecDIM system (circles) closely align with those measured by the commercial spectrometer (solid lines), demonstrating strong agreement. This consistency

confirms that the 3D-SpecDIM system able to accurately measure fluorescence spectra while providing the added advantage of high spatiotemporal resolution for single-molecule dynamics studies.

It should be noted that although careful calibration, the accuracy of spectral peak emission wavelength measurements can be affected by multiple factors. Differences in optical path alignment, calibration parameters, instrument detection characteristics, and environmental conditions such as viscosity, temperature, polarity, and pH can all induce slight spectral shifts.

2. Single molecule spectral tracking data acquisition

The real-time 3D single molecule tracking relies on real-time position estimation and active feedback control to maintain the target fluorophore within the excitation volume (**Supplementary Figure. 3**). Specifically, A pair of electro-optic deflectors (EODs) were used to deflect the laser focus in the XY plane, and a tunable acoustic gradient (TAG) lens was used to scan the laser focus in the Z axis. Fluorescence photons are collected by an avalanche photodiode (APD). The EODs were controlled with analogue voltage and the XY position of laser focus can be calculated by converting the voltage to distance. The TAG lens is employed to drive the laser focus in a sinusoidal motion along the axial direction. The laser focus position at any given time can be precisely calculated based on the output phase of the TAG lens. Upon detection of a fluorescence photon by the APD, its arrival time is used to correlate with the laser focus position, allowing for the estimation of the target molecule's position $\boldsymbol{p}_k$ with a field-programmable gate array (FPGA).

The position $\boldsymbol{p}_k$ is updated using a Kalman filter-based calculation, which integrates the prior position $\boldsymbol{p}_{k-1}$, the photon counts n_k, and the variance of the previous estimate σ_{k-1}^2. The formula for the updated position $\boldsymbol{p}_{k|k}$ is as follows:

$$\boldsymbol{p}_{k|k} = \frac{\boldsymbol{p}_{k|k-1} \cdot w^2 + c_k \cdot n_k \cdot \sigma_{k|k-1}^2}{w^2 + n_k \cdot \sigma_{k|k-1}^2}, \tag{7}$$

where w is the laser beam covariance, and c_k is the current laser position. The variance update is calculated by:

$$\sigma_{k|k}^2 = \frac{\sigma_{k|k-1}^2 w}{w^2 + n_k \cdot \sigma_{k|k-1}^2}. \tag{8}$$

The following equations provide predictions for the position and variance, respectively:

$$\begin{aligned} \boldsymbol{p}_{k|k} &= \boldsymbol{p}_{k-1|k-1}, \\ \sigma_{k|k-1}^2 &= \sigma_{k-1|k-1}^2 + 2D\tau, \end{aligned} \tag{9}$$

Where D is the expected diffusion coefficient and τ is the time between calculations (the bin time).

The workflow of the 3D-SpecDIM system is shown in **Supplementary Figure. 4**. For spectral acquisition, the camera settings must first be configured, including selecting the imaging region based on the tracked position, adjusting the cooling temperature, exposure time, and camera gain. Real-time EMCCD images are displayed through a custom-built LabVIEW program. Once tracking begins, the program automatically records and saves the spectral images until the tracking session concludes.

For the tracking process, the system initially drives the piezo to locate particles with fluorescence photon counts exceeding a predefined threshold. Upon capturing a single particle, the system updates the positional estimates (x_k, y_k, z_k) and converts these estimates into voltage signals to drive the piezo stage, ensuring the particle's position estimate is close to zero. It worth to note that the positional estimates (x_k, y_k, z_k) indicate the distances of molecule to the center of excitation volume in each direction. If the photon count falls below a

certain threshold, tracking is interrupted. This threshold is typically set at twice the background photon count to maintain tracking stability.

3. Description for custom-build 3D-SpecDIM LabVIEW program.

The 3D-SpecDIM LabVIEW program was developed based on the 3D-SMART program previously established by Prof. Kevin Welsher's lab. The primary modifications include configuring the camera parameters and synchronizing the EMCCD camera with the tracking system. The workflow of the program is illustrated in **Supplementary Figure. 4**

Supplementary Notes 4. Multi-resolution imaging with 3D-SpecDIM.

To reconstruct a 3D volumetric image of the mitophagy process, we employed two avalanche photodiodes (APDs) to simultaneously collect signals from mitochondria and lysosomes. Specifically, a 600/50 filter (FF01-600/52, Semrock) was positioned in front of APD1 (as shown in **Supplementary Fig. 1**) to enable active tracking based on HaloTag-JF549 signals. A 706/95 filter (ET706/95, Chroma) was placed in front of APD2 to capture lysosomal signals. The photon addressable capability of 3D-SpeDIM facilitates us to reconstruct the 3D image that surrounding target particle by registering the fluorescence photons to laser focus positions in a separated detection channel. Here the 3D image of lysosome was reconstructed in APD2. The recorded data included EOD scanning coordinates (XY information) and TAG phase (Z information) corresponding to the arrival of each photon. During data processing, we binned the photon counts at 100 ms intervals and allocated them into a 3D data voxel of dimensions 5×5×10, based on their coordinate values. When there is an overlap of EOD and TAG scanning voxel units at different trajectory points, we use the value with the higher photon count as the reconstruction vaule. Finally, by integrating these 3D data cubes with the 3D tracking trajectory, the 3D distribution of lysosomal morphology along the trajectory can be reconstructed. The 3D visualization of multi-resolution image was performed with Amira software.

Supplementary Notes 5. Calculation of diffusion coefficient.

The diffusion coefficient is derived by fitting the MSD curve to the following relationship:

$$MSD = 2nDt, \quad (10)$$

where n is the dimensionality of diffusion (in our case, n=3), t is the time interval, and D is the diffusion coefficient. According to the Einstein-Stokes equation:

$$D = \frac{k_B T}{6\pi\eta r}, \quad (11)$$

where k_B is the Boltzmann constant, T is the absolute temperature, η is the dynamic viscosity of the medium, and r is the hydrodynamic radius of the molecule, we can calculate the hydrodynamic radius of the molecule.

Supplementary References

1. Lee J, *et al.* Versatile phenotype-activated cell sorting. *Science Advances* **6**, eabb7438 (2020).
2. Dosovitskiy A, *et al.* An image is worth 16x16 words: Transformers for image recognition at scale. *arXiv preprint arXiv:201011929*, (2020).
3. Zhang Y, Liu T, Long M, Jordan M. Bridging theory and algorithm for domain adaptation. In: *International conference on machine learning*). PMLR (2019).
4. Sha H, Li HY, Zhang YB, Hou SG. Deep learning-enhanced single-molecule spectrum imaging. *Apl Photonics* **8**, (2023).
