# Supplementary material for: Single molecule spectrum dynamics imaging with 3D target-locking tracking
Source: Nat Commun. 2025 Sep 30;16:8686. doi: 10.1038/s41467-025-63787-3 (PMC12485062; doi:10.1038/s41467-025-63787-3)
Supplement: Supplementary file 2 — Description of Additional Supplementary Files [file 41467_2025_63787_MOESM2_ESM.pdf]

# Description of Additional Supplementary Files

**Supplementary Movie 1:** Discern two particles switching events during real-time single-particle tracking with 3D-SpecDIM. The trajectory of yellow, fluorescent particle and green, fluorescent particle are coded with yellow color and blue color, respectively. The particle switching can be observed at approximately 51 seconds. The video is played at 5.5× speed.

**Supplementary Movie 2:** Multi-resolution imaging of mitophagy process. The 3D lysosome volume image is overlayed with spectrally encoded 3D moving trajectory of mitochondrion. The trajectory's color represents the pH ratio. The right panel shows the real-time spectral images of the mGold-HaloTagJF549 probe. Lysosome intensity was reconstructed using Amira software and displayed using temperature-based color coding. The video is played at 6.4× speed.

**Supplementary Movie 3:** Time-lapse bright field movie of cellular blebbing imaging process. The cellular blebbing occurred at the periphery of the cell. The white arrow points to the tracked AgNP particle. The video is played at 52.5× speed.

**Supplementary Movie 4:** Spectral tracking of 200 nm fluorescent bead in water solution. The color of the trajectory denotes the time. The video is played at 0.8× speed.

**Supplementary Movie 5:** Spectral tracking of single setau647 dye molecule in 90% glycerol solution. The color of the trajectory
