# Supplementary material for: Single molecule spectrum dynamics imaging with 3D target-locking tracking
Source: Nat Commun. 2025 Sep 30;16:8686. doi: 10.1038/s41467-025-63787-3 (PMC12485062; doi:10.1038/s41467-025-63787-3)
Supplement: Supplementary file 8 — Reporting summary [file 41467_2025_63787_MOESM8_ESM.pdf]

nature portfolio

Corresponding author(s): Shangguo Hou

Last updated by author(s): Aug 5, 2025

Reporting Summary

Nature Portfolio wishes to improve the reproducibility of the work that we publish. This form provides structure for consistency and transparency in reporting. For further information on Nature Portfolio policies, see our Editorial Policies and the Editorial Policy Checklist.

Statistics

For all statistical analyses, confirm that the following items are present in the figure legend, table legend, main text, or Methods section.

n/a	Confirmed	
☐	☒	The exact sample size (*n*) for each experimental group/condition, given as a discrete number and unit of measurement
☐	☒	A statement on whether measurements were taken from distinct samples or whether the same sample was measured repeatedly
☒	☐	The statistical test(s) used AND whether they are one- or two-sided<br>*Only common tests should be described solely by name; describe more complex techniques in the Methods section.*
☒	☐	A description of all covariates tested
☒	☐	A description of any assumptions or corrections, such as tests of normality and adjustment for multiple comparisons
☐	☒	A full description of the statistical parameters including central tendency (e.g. means) or other basic estimates (e.g. regression coefficient) AND variation (e.g. standard deviation) or associated estimates of uncertainty (e.g. confidence intervals)
☒	☐	For null hypothesis testing, the test statistic (e.g. *F*, *t*, *r*) with confidence intervals, effect sizes, degrees of freedom and *P* value noted<br>*Give P values as exact values whenever suitable.*
☒	☐	For Bayesian analysis, information on the choice of priors and Markov chain Monte Carlo settings
☒	☐	For hierarchical and complex designs, identification of the appropriate level for tests and full reporting of outcomes
☒	☐	Estimates of effect sizes (e.g. Cohen's *d*, Pearson's *r*), indicating how they were calculated

Our web collection on statistics for biologists contains articles on many of the points above.

Software and code

Policy information about availability of computer code

Data collection	Microscope images were acquired using Dragonfly Confocal Microscopy System (Four/Seven-laser); Zeiss LSM 980 and Nikon Confocal Laser Scanning Microscope. The fluorescence emission spectrum was measured by BioTek Synergy H1;FS5, Edinburgh Instruments and Lumina, Thermo Scientific. The trajectory and spectral data were acquired by the homemade 3D-SpecDIM(3D target-locking-based single-molecule fluorescence Spectral Dynamics Imaging Microscopy) as described in manuscript.
Data analysis	FIJI (ImageJ win64) was used for confocal microscope image analysis. Python and matlab was used for trajectory and spectral analysis. The GitHub web link for the custom code developed in the study is https://github.com/houlab/3D-SpecDIM

For manuscripts utilizing custom algorithms or software that are central to the research but not yet described in published literature, software must be made available to editors and reviewers. We strongly encourage code deposition in a community repository (e.g. GitHub). See the Nature Portfolio guidelines for submitting code & software for further information.

Data

Policy information about availability of data

All manuscripts must include a data availability statement. This statement should provide the following information, where applicable:

- Accession codes, unique identifiers, or web links for publicly available datasets
- A description of any restrictions on data availability
- For clinical datasets or third party data, please ensure that the statement adheres to our policy

The spectral imaging and 3D trajectory data generated in this study have been deposited in the Zenodo database under accession code 10.5281/zenodo.15367615. The dataset includes raw and processed spectral imaging data, 3D trajectory data, and associated analysis files sufficient to verify the results reported in this article. Source data for all figures are provided within the Zenodo repository.

Research involving human participants, their data, or biological material

Policy information about studies with human participants or human data. See also policy information about sex, gender (identity/presentation), and sexual orientation and race, ethnicity and racism.

Reporting on sex and gender	N/A
Reporting on race, ethnicity, or other socially relevant groupings	N/A
Population characteristics	N/A
Recruitment	N/A
Ethics oversight	N/A

Note that full information on the approval of the study protocol must also be provided in the manuscript.

Field-specific reporting

Please select the one below that is the best fit for your research. If you are not sure, read the appropriate sections before making your selection.

☒ Life sciences ☐ Behavioural & social sciences ☐ Ecological, evolutionary & environmental sciences

For a reference copy of the document with all sections, see nature.com/documents/nr-reporting-summary-flat.pdf

Life sciences study design

All studies must disclose on these points even when the disclosure is negative.

Sample size	No statistical methods were used to predetermine sample size. Sample sizes for all experiments were based on prior publications in the field and established practices in super-resolution microscopy and single-molecule tracking, ensuring adequate statistical power to detect the reported effects. For trajectory-based analyses, the number of independent trajectories (n) was determined by collecting data until consistent statistical distributions and parameter convergence were observed across biological replicates. For confocal image experiment, at least three fields of view were captured per condition to sufficiently calculate statistics.
Data exclusions	For tracking experiment, the duration times of tracking was at least more than 10 seconds.
Replication	All experimental conclusion were successfully replicated with multiple independent experimental.
Randomization	Allocation of samples into experimental groups was determined by predefined experimental conditions (e.g., treatment vs. untreated control) rather than randomization. For each condition, multiple independent cells or samples were imaged under identical settings to control for potential covariates. Randomization was not relevant to this study because imaging fields and acquisition parameters were fixed and applied consistently across all samples.
Blinding	The investigator was not blinded as machine-based measurements of optical physical and fluorescence chemical properties are usually unaffected by knowledge of the sample.

Reporting for specific materials, systems and methods

We require information from authors about some types of materials, experimental systems and methods used in many studies. Here, indicate whether each material, system or method listed is relevant to your study. If you are not sure if a list item applies to your research, read the appropriate section before selecting a response.

Materials & experimental systems

n/a	Involved in the study
☒	☐ Antibodies
☐	☒ Eukaryotic cell lines
☒	☐ Palaeontology and archaeology
☒	☐ Animals and other organisms
☒	☐ Clinical data
☒	☐ Dual use research of concern
☒	☐ Plants

Methods

n/a	Involved in the study
☒	☐ ChIP-seq
☒	☐ Flow cytometry
☒	☐ MRI-based neuroimaging

Eukaryotic cell lines

Policy information about cell lines and Sex and Gender in Research

Cell line source(s)	Hela cell line was obatained from ATCC product (CCL-2). (https://www.atcc.org/products/ccl-2)
Authentication	*Describe the authentication procedures for each cell line used OR declare that none of the cell lines used were authenticated.*
Mycoplasma contamination	Not mycoplasma was detected in this study
Commonly misidentified lines (See ICLAC register)	No misidentified cell line was used in this study

Plants

Seed stocks	N/A
Novel plant genotypes	N/A
Authentication	N/A
